# Metabolic reprogramming in macrophages: the central role of lipid metabolism in bone homeostasis

**DOI:** 10.1038/s41413-026-00590-w

**Published:** 2026-10-30

**Authors:** Longping Li, Yuxiao Zhang, Shuo Huang, Yang Xiao, Genyu Liu, Zhengjie Wu, Yiqian Hu, Shiqiang Gong, Jing Mao, Ke Song, Qilin Li

**Affiliations:** 1 Department of Stomatology, Tongji Hospital, Tongji Medical College, Huazhong University of Science and Technology, Wuhan, PR China; 2 School of Stomatology, Tongji Medical College, Huazhong University of Science and Technology, Wuhan, PR China; 3 Hubei Province Key Laboratory of Oral and Maxillofacial Development and Regeneration, Wuhan, PR China

**Keywords:** Bone, Fat metabolism

## Abstract

Macrophage lipid metabolism is a pivotal regulator of bone homeostasis. Macrophages orchestrate the balance between bone resorption and formation primarily through their M1/M2 polarization states, with M1 macrophages promoting resorption and M2 macrophages supporting formation. This review comprehensively describes how key lipid metabolic pathways, such as fatty acid oxidation (FAO) and cholesterol metabolism, govern this polarization and subsequent bone remodeling. It integrates the core mechanisms by which lipid metabolism drives M1 and M2 polarization, and highlights that targeting macrophage lipid metabolism represents a promising therapeutic strategy for bone diseases. Finally, we identify emerging frontiers, including the “gut-bone axis” and epigenetic regulation, as critical directions for future research.

## Introduction

Bone homeostasis is the physiological balance between bone formation and resorption, ensuring stable bone mass, structure, and function, and is essential for skeletal health.^1^ In the context of bone homeostasis, macrophages significantly influence the osteoblast activation and osteoclast differentiation and contribute to the regulation of bone metabolism via inflammatory pathways.^2^ Bone tissue macrophages consist of various subtypes that collaborate in bone formation, resorption, and repair. M1 macrophages enhance osteoclast differentiation, increase inflammation, and speed up bone resorption. Conversely, M2 macrophages aid osteoblast differentiation, suppress osteoclast formation, and possess anti-inflammatory properties, thereby promoting the repair of inflammatory bone damage and bone formation after fractures.^3^

Lipid metabolism processes, including the metabolism and accumulation of fatty acids and cholesterol in macrophages, have the potential to induce metabolic reprogramming, which can subsequently influence macrophage polarization and the secretion of inflammatory mediators via cellular signaling pathways such as NF-κB and Wnt.^4,5^ Lipid metabolism supplies energy for macrophage activation and influences their polarization into M1 and M2 types through distinct pathways, with various lipid molecules affecting the polarization process differently.^6^ Fatty acid and cholesterol metabolism shape macrophage phenotypes in a context-dependent manner. Excessive saturated fatty-acid uptake and intracellular cholesterol overload generally favor inflammatory activation, whereas efficient cholesterol efflux and pro-resolving lipid pathways help restore lipid homeostasis and limit inflammation; fatty acid oxidation (FAO) and lipid storage may support reparative phenotypes under appropriate stimuli.^7^ Furthermore, distinct fatty acids exert diverse regulatory influences on macrophage functions. For instance, 12-hydroxyeicosatetraenoic acid can promote the pro-inflammatory phenotype of macrophages, promoting osteoclast differentiation and aggravating bone loss, while short-chain fatty acids (SCFAs) can inhibit this process and instead promote osteoblast differentiation and osteogenic activity.^8,9^

Macrophage lipid metabolism influences their polarization and bioactive substance secretion, playing a role in bone homeostasis regulation.^10,11^ Activated M1 macrophages promote osteoclast differentiation and bone resorption by secreting macrophage colony-stimulating factor (M-CSF) and receptor activator of nuclear factor-κB ligand (RANKL), while also inducing inflammation in the surrounding bone environment, further increasing bone resorption. In contrast, activated M2-type macrophages, while having an anti-inflammatory phenotype, can inhibit osteoclast differentiation and promote osteoblast differentiation through signaling pathways such as Wnt/β-catenin. Disrupted lipid metabolism in macrophages can impair their function and polarization, affecting bone homeostasis. Targeting lipid metabolism, particularly via the SHBG-SREBP1 axis, can alter macrophage polarization, decrease inflammation, and enhance bone regeneration.^12^ Abnormal lipid metabolism can disrupt M1 and M2 macrophage polarization, leading to an imbalance between osteoclasts and osteoblasts and impacting bone homeostasis. Elevated oxidized lipids and cholesterol can induce M1 polarization via inflammatory and oxidative stress pathways. Similarly, in osteoarthritis, disrupted lipid metabolism worsens joint degeneration by enhancing inflammation.^13^

This review examines how lipid metabolism influences macrophage roles in bone homeostasis, detailing macrophage biology, regulatory mechanisms, and the impact of lipid metabolism on their function. It explores the dual roles of macrophage lipid metabolism in inflammatory bone diseases and bone remodeling, highlighting therapeutic potential and future applications.

## Functional plasticity and regulatory mechanisms of macrophages in bone homeostasis

### Molecular mechanisms of macrophage polarization: from M1 to M2 states

Macrophage differentiation undergoes two waves of blood cell development processes.^14^ In embryos, macrophages originate from primitive macrophages. In the third week of pregnancy, blood islands release primitive macrophages after the establishment of blood circulation. These primitive macrophages then migrate throughout the body via the bloodstream and colonize various tissues.^15,16^ Erythro-myeloid progenitors in hemogenic endothelium migrate to the fetal liver through the blood circulation, generating fetal monocytes. These monocytes enter the blood, colonize tissues other than the brain, and gradually differentiate into tissue-resident macrophages (RTMs).^17,18^ Adult monocytes are mainly derived from the bone marrow.^19^ By the end of the 5th week of gestation, hemogenic endothelial cells produce the first hematopoietic stem cells (HSCs), which then undergo hematopoiesis to form monocytes, aiding in the renewal of macrophages and lymphocytes.

**Fig. 1:**
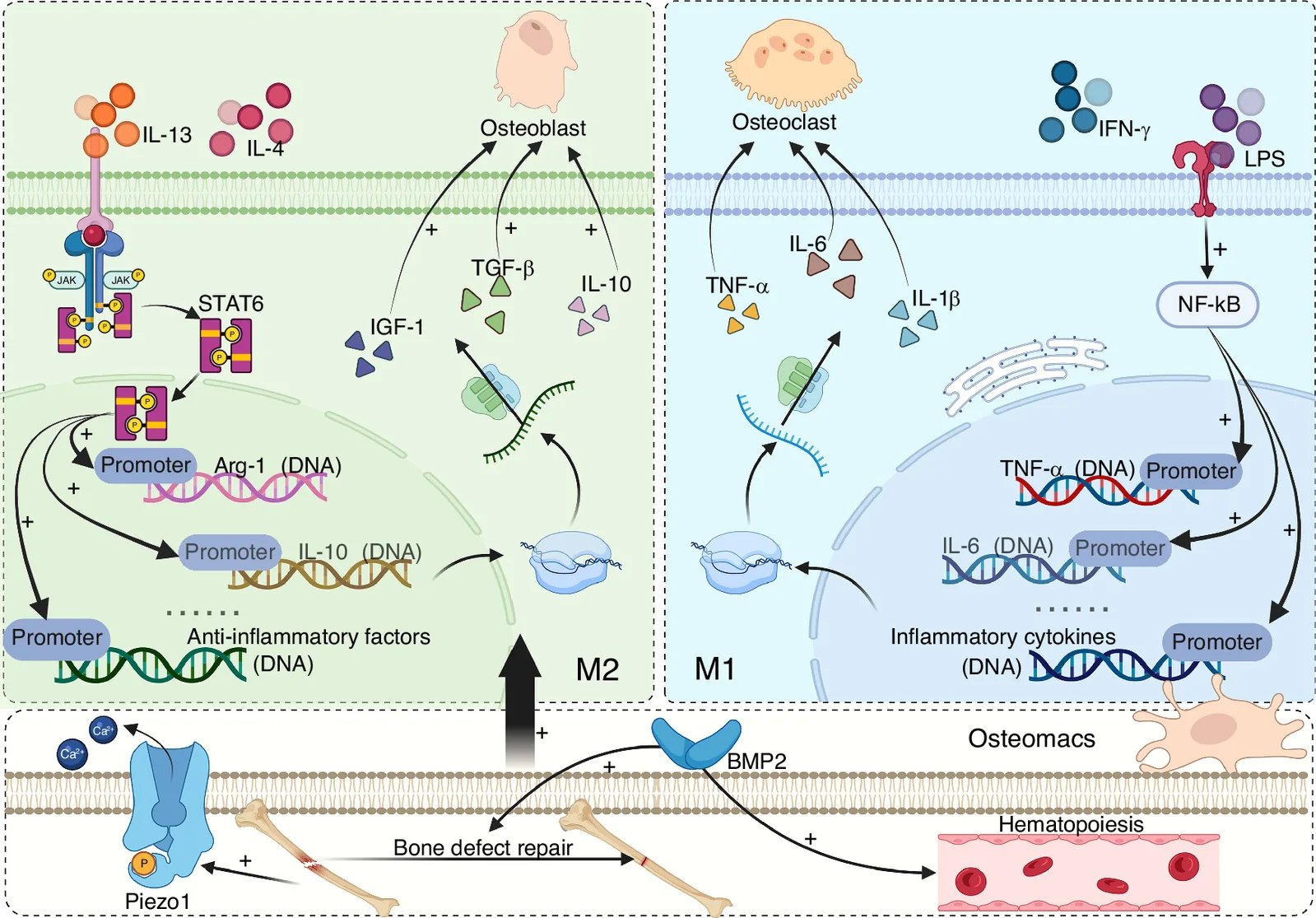
Polarization of macrophages in inflammation and bone repair. IL-4/IL-13 induce M2 polarization through JAK-STAT6, and the expressed IGF-1, TGF-β, and IL-10 can promote the proliferation and differentiation of osteoblasts. LPS/IFN-γ trigger M1 polarization through NF-κB, induce the production of TNF-α, IL-1β, and IL-6, and can promote osteoclast differentiation. Osteomacs regulate hematopoiesis and repair processes via BMP2, and promote bone formation by activating Piezo1 through mechanical stimulation

Macrophages have a high degree of adaptability. In the classical classification, macrophage subtypes are divided into M1 and M2 types. Inflammatory factors like lipopolysaccharide (LPS) and IFN-γ induce macrophages to polarize into the M1 type. Pattern recognition receptors like Toll-like receptors (TLRs) detect pathogen-associated molecular patterns such as LPS, triggering the NF-κB signaling pathway, starting the transcription of inflammatory cytokines like tumor necrosis factor-α (TNF-α), Interleukin-1β (IL-1β), and Interleukin-6 (IL-6), leading macrophages to adopt pro-inflammatory M1 characteristics.^20^ In the presence of cytokines IL-4 and IL-13, macrophages become M2 type. These cytokines activate the JAK-STAT6 pathway, influencing genes like arginase-1, Ym1, and IL-10, which induce M2 characteristics in macrophages^21,22^ (Fig. 1). In classical classification, scholars have divided macrophages into pro-inflammatory (M1 type) and anti-inflammatory/repairing (M2 type) based on the above in vitro induction, as well as the expression of key surface markers and the production of cytokines. However, it is generally believed that the M1/M2 dichotomy paradigm of macrophage polarization is overly simplified and cannot accurately explain macrophage phenotypes in different contexts. Currently, there is no standardized nomenclature that can fully cover the diversity of macrophage origins, stimulating factors, and microenvironments. Although M1-type and M2-type polarized macrophages represent two extremes of a continuous activation state, in this review, we still adopt the M1/M2 dichotomy to define the polarity of macrophages in order to understand the complex plasticity of macrophages.

Macrophage polarization is influenced by various molecules. For instance, STAT6 antisense oligonucleotides can suppress STAT6, converting M2 to M1 phenotype;^23^ 4-octyl itaconate (OI) can inhibit the phosphorylation of STAT6 and JAK1, and suppress IL-4 induced metabolic reprogramming, thereby inhibiting M2 polarization.^24^ In contrast, in the tumor microenvironment (TME), Wnt ligands secreted by tumor cells can activate the Wnt/β-catenin signaling pathway in tumor-associated macrophages (TAMs), promoting the polarization of M2 macrophages.^25^ Wnt3a or LiCl can also activate this signal pathway.^26^ In addition, SIRT4 silencing promotes the polarization of TAM towards M2 through the FAO-PPARδ-STAT3 signaling pathway.^27^ Furthermore, the deletion of receptor-interacting protein kinase 3 (RIPK3) in TAMs reprograms fatty acid metabolism through the ROS-caspase1-PPAR pathway, promoting M2 polarization.^28^

Macrophages interact with osteoblasts and osteoclasts through direct cell-cell contact or paracrine mechanisms. The effect of macrophages on osteocytes depends on their polarization ratio and the paracrine factors they secrete.^29^ M1 macrophages release inflammatory cytokines like TNF-α, IL-1β, and IL-6, stimulating osteoclast precursor cells to mature. They also secrete osteopontin, which activates osteoclasts to degrade bone matrix.^30^ M2 macrophages secrete anti-inflammatory cytokines like IL-10, which curb inflammation and protect bone tissue.^31^ They also release growth factors such as insulin-like growth factor-1 (IGF-1) and transforming growth factor-β (TGF-β) to enhance osteoblast proliferation and differentiation, while regulating osteoclast activity to maintain bone homeostasis.^32^ Therefore, macrophage subtypes play a crucial role in bone metabolism.

### Macrophage subtypes and their roles in bone metabolism

In the skeletal system, maintaining bone health relies on the balance between bone resorption and formation, with macrophages playing a crucial role. Macrophage fusion and multinucleation are essential for osteoclast formation, which is regulated by factors like PPARγ, PGC-1β, NDUFS4, and VLDLR.^33^ Furthermore, macrophage metabolic disorders cause monocytes and macrophages to overly differentiate into osteoclasts, thereby triggering bone diseases.^34^ Bone marrow macrophages, like osteomacs, influence osteoblast function, impacting bone formation and hematopoiesis. Osteomacs are a “functionally specialized subtype” of macrophages that focus on bone metabolism and repair in bone tissue, belonging to tissue-resident macrophages.^35^ Osteomacs reside long-term on the surface of bone trabeculae and can exhibit M1 or M2 phenotypes to participate in bone metabolism in response to changes in the bone microenvironment (such as fractures, inflammation, and mechanical load). During bone injury or inflammation, Osteomacs polarize towards the M1 phenotype, while during bone repair or homeostasis maintenance, they polarize towards the M2 phenotype.^36^ Normally, these macrophages are subject to various regulations. For example, glucocorticoids control the activity of osteoblasts by enhancing the expression of bone morphogenetic protein 2 (BMP2);^37^ Piezo1 is a calcium channel regulated by mechanical force; When bones are damaged, mechanical stimulation of these macrophages can promote bone formation.^38^ In addition, macrophages help regulate the balance between bone resorption and formation by interacting with osteoclasts and osteoblasts. They secrete osteopontin (OPN), which promotes bone resorption and fat phagocytosis, but OPN also inhibits ATP6V0d2 expression, causing lactate buildup and reducing osteoclast production,^30^ forming a negative feedback loop to maintain the balance between osteoblasts and osteoclasts. The accumulation of OPN may promote the polarization of osteoclasts.^39^ Furthermore, studies have found that Fam20C, as a kinase, can phosphorylate OPN to reduce its secretion level, while OPN neutralization can reduce osteoclast differentiation and cancer bone metastasis induced by Fam20C deficiency.^40^

M1 macrophages promote inflammation and primarily contribute to bone resorption and destruction. They release pro-inflammatory cytokines and ROS, inhibiting osteoblast differentiation, and secrete M-CSF and RANKL to encourage osteoclast formation.^41,42^ RANKL is involved in key processes such as osteoclast differentiation, activity enhancement, and resorption. After the body is infected, macrophage polarization releases inflammatory factors and RANKL. RANKL binds to receptor activator of nuclear factor κB (RANK) to activate the NF-κB and MAPK pathways, promoting osteoclast differentiation and maintaining osteoclast circulation.^43,44^ In addition to RANKL being able to participate in regulation, Mei-hua Chen et al. found in a mouse periodontitis model that the hypoxia-inducible factor-1α (HIF-1α) activator dimethyloxalylglycine can inhibit the M1-like polarization of RAW264.7 macrophages and mouse bone marrow macrophages, and downregulate the expression of TNF-α, IL-6, CD86, and monocyte chemoattractant protein-1 (MCP-1), thereby achieving the effect of inhibiting bone resorption.^45^ M1 macrophages secrete TNF-α, IL-1, IL-6, etc., which can directly or indirectly promote the differentiation and formation of osteoclasts and enhance the bone resorption capacity of osteoclasts.^46^

M2 macrophages play an anti-inflammatory role and are of great significance in the protection and repair of bone tissue. M2 macrophages secrete anti-inflammatory and osteogenic factors, promote the differentiation of osteoblasts, inhibit the formation of osteoclasts, and accelerate bone formation.^47^ M2 macrophages can induce the Wnt/β-catenin signaling pathway in bone marrow mesenchymal stem cells, promote the expression of osteoblast-related genes, and then differentiate into osteoblasts.^48,49^ In addition, signaling molecules such as BMP2 and TGF-β1 secreted by macrophages regulate the gene expression of osteoblasts through SMAD proteins.^50,51^ This pathway is also regulated by other bioactive substances. For example, thyroid hormones affect the BMPs pathway, leading to hyperthyroidism-induced osteoporosis.^52^ In addition, TGF-β can not only regulate the differentiation of osteoblasts through SMAD but also regulate the Wnt signaling pathway through non-SMAD pathways.^53^ In addition, various cytokines secreted by macrophages, such as IL-1, IL-6, and TNF-α, regulate the activity and function of osteoblasts.^54^ In addition, bioactive substances such as platelet-derived growth factor (PDGF), vascular endothelial growth factor (VEGF), and PGE2 secreted by macrophages support the function of osteoblasts.^55^ PDGF can activate the expression of related target genes, which can promote the proliferation and differentiation of MPCs. Regenerative MPCs express bone regeneration-related genes (Runx2 and Msx2), which can promote the differentiation of osteoblasts.^56,57^ Secondly, after macrophages clear apoptotic cells, they can express VEGF. Through direct action or by promoting angiogenesis, it supports the metabolic and functional activities of osteoblasts and promotes bone formation.^58^ IL-1β secreted by macrophages can also regulate the transcription of pro-angiogenic VEGF through STAT3 and NF-κB.^59^ However, overexpression of VEGF will significantly increase the recruitment of osteoclasts.^60^ PGE2 released through connexin 43 (Cx43) hemichannels in osteocytes inhibits adipogenesis and promotes osteogenesis via PGE2 receptors.^61,62^

## Role of macrophage metabolic reprogramming in bone remodeling

Energy metabolism is an essential factor for macrophages to carry out life activities. Metabolic reprogramming can affect the polarization of macrophages, and macrophages with different phenotypes have different tendencies in energy metabolism. In M1-type macrophages, glycolysis is enhanced while FAO is inhibited.^63,64^ Glycolysis-derived pyruvate can be converted to acetyl-CoA, which is preferentially used for the synthesis of free fatty acids (FFA) rather than FAO. In M2 macrophages, FAO and OXPHOS are synergistically enhanced.^65–67^ The acetyl-CoA produced by FAO provides a continuous substrate for the TCA cycle, strengthening the energy production of OXPHOS.

During the process of pathological changes, enhanced glycolysis in macrophages promotes M1 macrophage polarization and inflammatory responses. Inflammatory FLSs secrete inflammatory exosomes, which macrophages absorb, activating HIF1A, increasing glycolytic enzyme expression, and promoting M1 polarization and inflammatory factor release.^68^ Activating KLF14 can suppress HK2 transcription by binding to its promoter, lower macrophage glycolytic activity, and reduce pro-inflammatory factor secretion.^69^ In the TME, macrophages experience glycolytic reprogramming, influenced by the “Warburg Effect,” which produces metabolites like lactic acid. Zeb1, a crucial regulator of glycolytic genes, is enhanced by hypoxia and promotes M2-type TAM polarization via the lactate-PKA/CREB pathway.^70^ In terms of bone regulation, aconitate decarboxylase 1 (Acod1) inhibits osteoclast differentiation by suppressing succinate dehydrogenase-dependent reactive oxygen species production and Hif1α-mediated aerobic glycolysis.^71^

Amino acid metabolism and its metabolites can participate in regulating the polarization and life activities of macrophages. For instance, citrulline can decrease inflammation and cellular aging by blocking the mTOR-HIF1α-glycolysis pathway in macrophages.^72^ In contrast, LPS stimulation triggers the NF-κB pathway, boosting EAAT2 expression, which facilitates M1 macrophage polarization via the lysosomal Glu/Asp transport-V-ATPase-mTORC1 pathway.^73^ Amino acid metabolic reprogramming can influence macrophage interactions with other cells. For instance, macrophage-induced C-type lectin (Mincle) activates the SYK/calcium pathway, enhancing osteoclast formation and activity.^74^ α-ketoglutarate (αKG) produced by glutamine metabolic decomposition activates FAO and Jmjd3-dependent epigenetic reprogramming of M2 genes by increasing the αKG/succinate ratio, while a low ratio promotes M1 polarization.^75^

Oxidative phosphorylation (OXPHOS) can affect the polarization of macrophages and has a selective regulatory role in the maintenance of tissue macrophage (TMFs) homeostasis.^76^ Celastrol binding to PKM2 suppresses the Akt/mTOR/HIF-1α pathway, reduces glycolytic enzyme expression, enhances mitochondrial OXPHOS, and shifts M1 macrophages towards M2 polarization by reversing the Warburg effect.^77^ PGAM5 expression is notably higher in OA synovial macrophages, where it dephosphorylates DVL2, suppresses the β-catenin pathway, and promotes M1 macrophage polarization.^78^ L-arginine promotes inflammatory osteoclasts to switch their glycolytic pathway to OXPHOS, thereby increasing ATP production, facilitating purine metabolism, elevating inosine and hypoxanthine levels, and inhibiting osteoclast formation.^79^

**Fig. 2:**
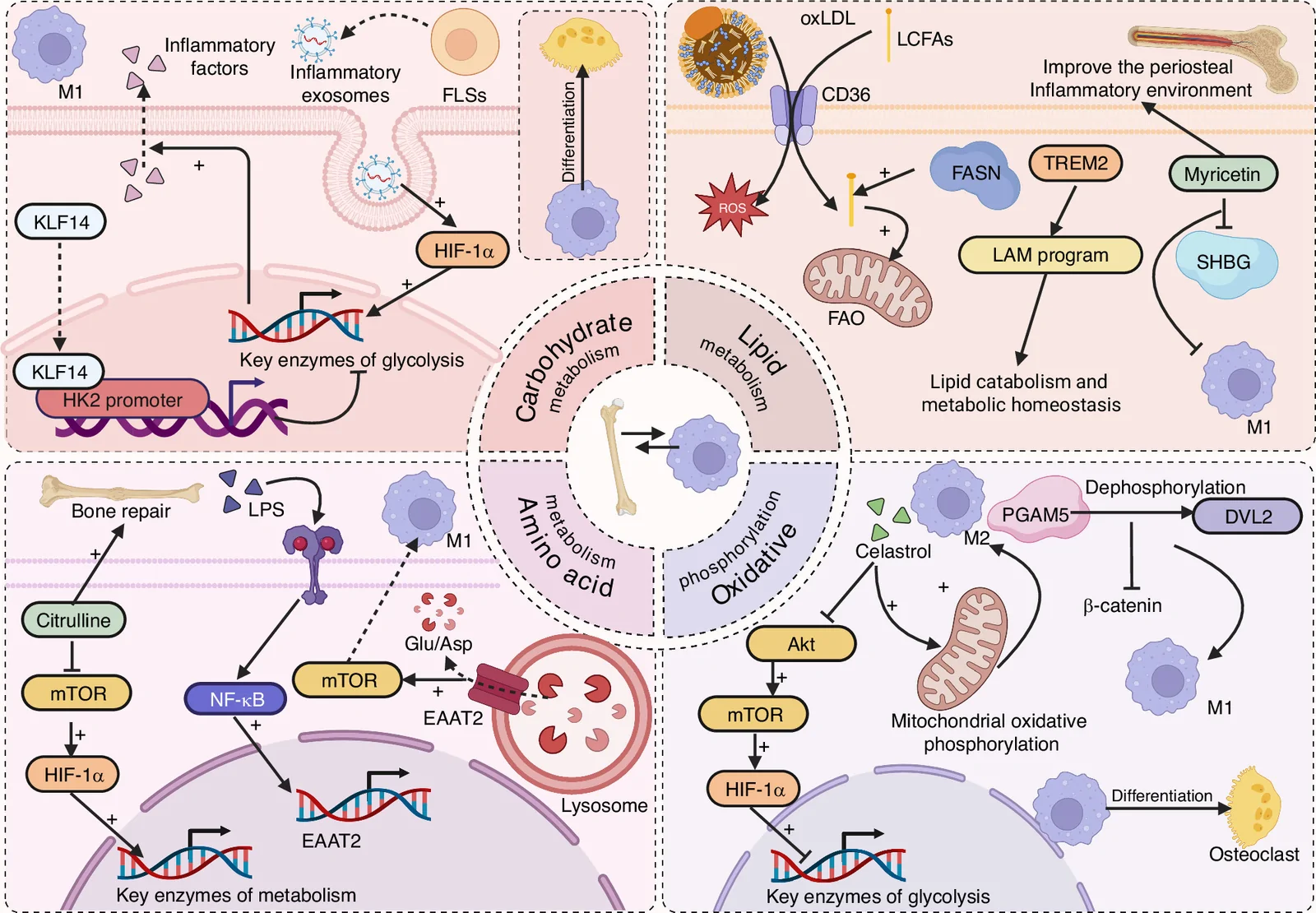
Metabolic regulatory network of macrophage polarization. Carbohydrate metabolism: KLF14 binds to the HK2 promoter to inhibit glycolysis, whereas inflammatory exosomes activate HIF-1α-dependent glycolysis and promote M1 polarization. Lipid metabolism: oxLDL-CD36 signaling increases ROS production, whereas CD36-mediated uptake of LCFAs enhances FAO; TREM2 drives a lipid-associated macrophage program involved in lipid handling, lipid catabolism, and energy metabolism, thereby contributing to metabolic homeostasis. Amino acid metabolism: LPS activates NF-κB and increases EAAT2 expression, whereas citrulline inhibits the mTOR-HIF-1α-glycolysis axis and attenuates inflammatory activation. Oxidative phosphorylation: Celastrol suppresses the Akt-mTOR-HIF-1α pathway and promotes a shift from M1 toward M2 polarization, whereas PGAM5 promotes M1 polarization through DVL2/β-catenin signaling

Lipid metabolism reprogramming in macrophages is key to controlling their polarization and functions. Enhanced lipid metabolism boosts macrophage inflammation. For instance, in inflammatory settings, oxidized low-density lipoprotein (LDL) interacts with CD36, leading to reactive oxygen species production and inflammation. Moreover, CD36-mediated uptake of LCFAs in the TME can enhance FAO and promote a tumor-supportive macrophage phenotype.^80^ Moreover, fatty acid synthase (FASN) can lead to FFA polarizing macrophages into a TAM-like phenotype, creating an immunosuppressive microenvironment.^81^ Molecular characteristics of TAMs were identified through single-cell RNA sequencing (scRNA-seq) data analysis. Dipeptidyl peptidase VII (DPP7) acts as a key gene in TAMs. DPP7 reduces ubiquitination-induced degradation of carnitine palmitoyltransferase 1A (CPT1A), thereby enhancing FAO and further inducing M2 polarization.^82^ TREM2-dependent lipid-associated macrophages support lipid handling and metabolic homeostasis by activating transcriptional programs related to phagocytosis, lipid catabolism, and energy metabolism.^83^ Regulating macrophage lipid metabolism may become a therapeutic strategy for bone-related diseases. For example, Myricetin prevents M1 macrophage polarization by inhibiting SHBG, thereby improving lipid metabolism mediated by SREBP1.^84^ Autophagy activators (such as LEO) reduce lipid accumulation in macrophages by improving lipid metabolism profiles.^85^ Lipid metabolism in macrophages is crucial for bone homeostasis, and targeting it could be promising for treating bone diseases (Fig. 2).

## Lipid metabolism in macrophages from pathways to functional regulation

### Core pathways of lipid metabolism in macrophages

The bone remodeling unit is a functional unit that couples bone resorption (by osteoclasts) and bone formation (by osteoblasts).^86^ Its microenvironment is characterized by the enrichment of immune cells, gradients of cytokine concentrations, a unique metabolic microenvironment, and the presence of local immune regulatory barriers.^87^ Immune cell communities, dominated by macrophages, are enriched, forming an immune-osteocyte interaction network. Meanwhile, high-concentration gradients of cytokines/chemokines (such as M-CSF, RANKL, OPG, TNF-α, IL-1β, and TGF-β) are formed, which are not only regulators of bone metabolism but also key signals for immune cell polarization. Additionally, the local metabolic microenvironment, including hypoxia, lactic acid accumulation, and high concentrations of lipid metabolites (such as prostaglandins and oxidized phospholipids), regulates the metabolic reprogramming and functional polarization of macrophages. This microenvironment does not isolate immune responses but screens and regulates the functions of immune cells, enabling macrophages to deviate from the classical immune phenotypes in the periphery and transform into phenotypes adapted to bone metabolism.^88–90^

#### Fatty acid oxidation (FAO) mechanisms and regulatory networks

FAO consists of steps such as fatty acid uptake, activation, and transport into mitochondria. Macrophages acquire fatty acids from the surrounding environment, with these lipids originating from plasma lipoproteins (e.g., LDL and VLDL) or extracellular-matrix fatty-acid-binding proteins.

**Fig. 3:**
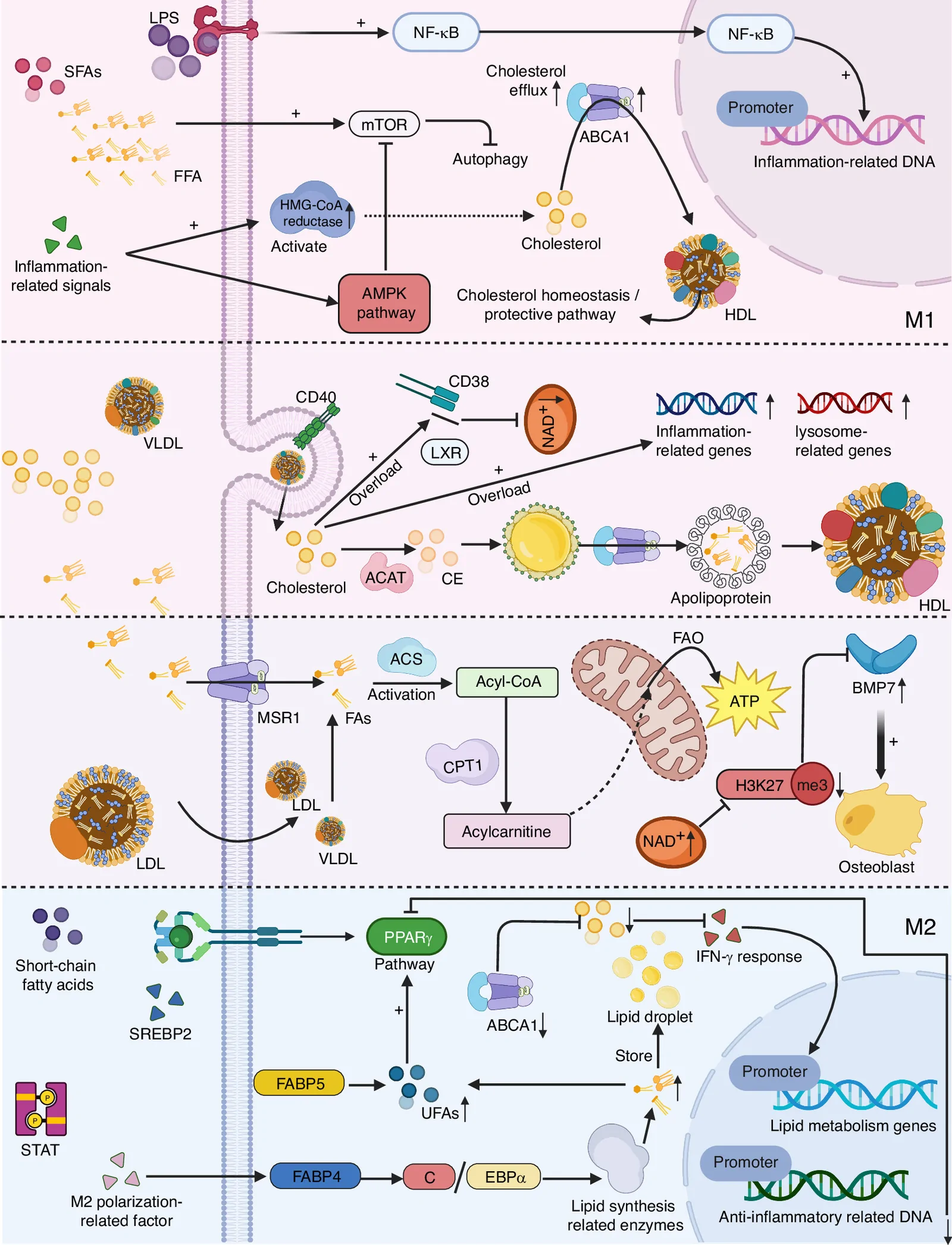
The association between macrophage M1/M2 polarization and lipid metabolism, inflammation, and bone regulation. During M2 polarization, factors mediated by STAT and others play a role. The FABP4/5 and PPARγ pathways are involved in promoting the expression of lipid synthases and regulating anti-inflammatory and lipid metabolism genes; they also promote BMP-mediated osteogenesis through NAD^+^, H3K27 methylation, etc. During the M1 polarization process, FFAs activate the AMPK and mTOR pathways, affecting cholesterol efflux. Meanwhile, cholesterol overload can regulate inflammatory genes through CD40 and LXR

Macrophages take up exogenous fatty acids (FAs) through macrophage scavenger receptor 1 (MSR1), which promotes macrophage FAO and OXPHOS to supply energy for cells and increases NAD⁺ levels, thereby activating silent information regulator 1 (SIRT1), reducing the acetylation level of enhancer of zeste homolog 2 (EZH2), decreasing H3K27me3 modification, promoting BMP7 expression, and further promoting the osteogenic differentiation of bone marrow mesenchymal stem cells (BMSCs).^91,92^ Under inflammatory conditions, macrophages usually produce lipid accumulation after taking up fatty acids, which in turn leads to lesions. However, after treatment with obeticholic acid, it can inhibit the activation of the NOD-like receptor pyrin domain-containing protein 3 (NLRP3) inflammasome in macrophages and regulate the lipid accumulation caused by the activation of the inflammasome.^93^ The ingested fatty acids need to be catalyzed by acyl-CoA synthetase (ACS) to form acyl-CoA before FAO. Acyl-CoA is transported into mitochondria through carnitine palmitoyltransferase 1 (CPT1) for β-oxidation.^94^ During this process, it is regulated by the CD40 signal. After CD40 activation, macrophages initiate FAO. The acetyl-CoA produced by FAO regulates histone acetylation through ATP-citrate lyase (ACLY), thereby supporting FAO and OXPHOS.^95^ Moreover, fatty acid metabolism is related to the PPARs signaling pathway. RIPK3 can promote fatty acid metabolism by activating the PPARs signaling pathway, inhibiting TAM polarization^28^ (Fig. 3).

FAO is modulated by multiple factors, including hormones, nutritional status, intracellular signaling pathways, and various bioactive or chemical compounds. For example, insulin can inhibit FAO, while glucagon and adrenaline can promote FAO; FAO is activated when cells are short of energy to provide more energy. Moreover, Zhexu Chi et al. reported that histone deacetylase 3 (HDAC3) restricts exogenous fatty acid-mediated FAO and OXPHOS; After knockout or inhibition of HDAC3 in myeloid cells, the levels of long-chain acylcarnitine in macrophages decrease, while acetylcarnitine levels increase, and fatty acid uptake is elevated (CD36 expression is upregulated), thereby promoting FAO.^96^ Under pathological conditions, mitochondrial STAT3 modulates FAO and contributes to disease progression.^97^ Furthermore, pharmacological agents such as the FAO inhibitor etomoxir (ETO), the fatty acid synthase inhibitor TOFA, and the lipolysis inhibitor orlistat can all participate in regulating fatty acid metabolism in macrophages.^28^ In summary, FAO is essential for maintaining macrophage energy balance and functional adaptation. Its regulation integrates metabolic and immune signaling, linking energy production to macrophage polarization and inflammatory activity.

#### Cholesterol metabolism

Cholesterol metabolism is an important part of macrophage metabolism, consisting of steps such as cholesterol uptake, oxidation, and efflux. Macrophages can take up cholesterol through multiple pathways, with the most important one being endocytosis mediated by the LDL receptor. During this process, NO2-OA can interact with CD36, inhibiting the binding and uptake of mLDL to CD36.^98^ Ingested cholesterol can be catalyzed by Acyl-CoA: cholesterol acyltransferase (ACAT) in cells to form cholesterol esters (CE). In addition, it can also be catalyzed into 27-hydroxycholesterol (27HC) by cholesterol-27-hydroxylase (cyp27a1). 27HC can bind to estrogen receptor α (Erα), leading to the dissociation of Septin 11 and the activation of the JNK signaling pathway.^99^ Esterified cholesterol can be stored in lipid droplets to prevent cholesterol-induced cytotoxicity. Over time, these lipid droplets undergo metabolism and are excreted via reverse cholesterol transport in the liver. This process involves several key proteins, including ATP-binding cassette transporters ABCA1 and ABCG1, as well as apolipoprotein A-I (apoA-I). ABCA1 mediates the transport of intracellular cholesterol to apoA-I to form nascent high-density lipoprotein (HDL) particles and can also transfer cholesterol to mature HDL. Moreover, multiple autophagy-related genes such as Map1lc3a and Tfeb, along with lipid droplet–associated proteins such as Hmgb1 and Hmgb2, have been shown to regulate this process.^100^ APOA1-mediated cholesterol efflux can protect mitochondrial function, and thereby enhance macrophage phagocytosis.^101^ Under pathological conditions, impaired cholesterol efflux can lead to cholesterol accumulation within the tissue microenvironment.^102^ Conversely, abnormal efflux and cholesterol overload can deplete NAD⁺ in macrophages through the LXR/CD38 signaling pathway, ultimately inducing macrophage senescence.^103^ In addition, retained cholesterol crystals within macrophages can activate inflammatory pathways, upregulate inflammatory cytokines and lysosome-related genes, damage lysosomal membranes, and induce the activation of early growth response factor 1 (Egr1)^104^ (Fig. 3).

Cholesterol metabolism is further modulated by multiple factors, including intracellular cholesterol concentration, hormones, signaling pathways, and various chemical compounds. Macrophages upregulate Ch25 to inhibit cholesterol synthesis and activate sterol regulatory element-binding protein (SREBP), which regulates the expression of genes related to cholesterol synthesis and uptake to maintain homeostasis.^105^ Meanwhile, the lipid-peroxidizing enzyme arachidonate 15-lipoxygenase type B (ALOX15B) also influences cholesterol metabolism. Silencing of ALOX15B reduces ERK1/2 phosphorylation, thereby decreasing nuclear sterol regulatory element-binding protein 2 (SREBP2) levels, leading to a decrease in cholesterol biosynthesis intermediates and oxysterols, along with a reduction in lipid peroxidation levels and the levels of 15-lipoxygenase-specific lipoxins.^106^ Furthermore, Guobin Zheng et al. demonstrated that Ganoderma lucidum spore powder (GLSP) enhances cholesterol efflux by upregulating ABCA1 and ABCG1 expression. At the same time, GLSP modulates genes involved in lipid metabolism by promoting lipid catabolism and suppressing lipid synthesis, thereby markedly reducing lipid accumulation in macrophages and inhibiting their transformation into foam cells.^107^

### The role of lipid metabolism in macrophage activation and polarization

#### Fatty acid and cholesterol metabolism coordinately regulate M1 macrophage polarization

The uptake and intracellular accumulation of fatty acids can promote the M1 polarization of macrophages. For example, upregulation of the membrane transport protein ABCG1 affects the distribution of fatty acids, causing fatty acids to redistribute from lipid droplets to membrane phospholipids. The content of saturated fatty acids (SFAs) in membrane phospholipids increases, while the reduction of SFA in lipid droplets, thereby enhancing the inflammatory response.^108^ Once internalized, fatty acids undergo β-oxidation to provide energy that supports the activation and effector functions of M1 macrophages. Furthermore, high concentrations of FFA can inhibit mTOR-mediated autophagy, leading to a significant increase in the mean fluorescence intensity (MFI) of M1 markers (CD86, iNOS) in monocyte-derived macrophages, and activating the NF-κB pathway, thereby causing a secondary increase in pro-inflammatory markers.^109^ Excessive uptake of exogenous fatty acids and their intracellular accumulation can promote FAO in macrophages. However, impaired OXPHOS inhibits FAO, while enhanced glycolysis produces lactic acid, which promotes the polarization of macrophages toward the M1 phenotype.^110^ Inflammatory stimuli can increase cholesterol synthesis by upregulating enzymes such as HMG-CoA reductase. By contrast, ABCA1/ABCG1-dependent cholesterol efflux is a compensatory homeostatic response that limits cholesterol overload rather than a general driver of M1 polarization. Lipophagy supports the mobilization of cholesterol for efflux,^100^ while 13-series resolvin (RvT)4 promotes neutral cholesterol ester hydrolase (nCEH)-dependent neutral-lipid hydrolysis and Scavenger Receptor class B type 1 (SR-BI) translocation, thereby enhancing cholesterol efflux and favoring an inflammation-resolving phenotype.^111^ Tim4⁺Lyve1⁺ resident adipose-tissue macrophages couple lysosomal lipid processing to ABCA1-dependent HDL release; however, this study addressed postprandial cholesterol transport rather than M1 polarization^112^ (Fig. 3).

#### Lipid synthesis and storage promote M2 macrophage polarization

Different metabolisms of fatty acids play an important regulatory role in macrophage polarization. Compared with M1 macrophages, M2 macrophages tend to accumulate endogenous lipids, thereby reducing intracellular FFA and enhancing lipid breakdown, which promotes FAO and makes the cells exhibit an anti-inflammatory phenotype. In macrophages, the FABP4/C/EBPα pathway can activate lipid synthesis-related enzymes such as fatty acid synthase, promote the formation of triglycerides (TAG) and lipid droplets, and at the same time, FABP4 further mediates lipolysis.^113^ These synthetic lipids can be used to construct cell membranes or form LDs to store energy, providing a material basis for M2 macrophages to perform functions such as tissue repair. FABP5 can selectively regulate the metabolism of long-chain unsaturated fatty acids (UFAs) to control the polarization of macrophages. In FABP5-KO macrophages, the levels of long-chain UFAs (such as oleic acid, arachidonic acid, and linoleic acid) are significantly increased, the peroxisome proliferator-activated receptor γ (PPARγ) signaling pathway is activated, and the expression of genes related to fatty acid β-oxidation, tricarboxylic acid cycle (TCA), and OXPHOS in macrophages is upregulated, thereby promoting M2 polarization.^114^ Macrophages can also take up these fatty acids through CD36, drive fatty acid β-oxidation, enhance mitochondrial respiration, and induce M2 polarization.^115^ In addition, scholars have also found in Mycoplasma gallisepticum infection models that quercetin (QUE) can induce FAO via the PI3K/AKT pathway, thereby promoting M2‑type polarization.^116^ It is inferred from this that UFAs can promote M2 polarization by promoting genes related to lipid oxidative metabolism and inhibiting genes related to anabolic metabolism. The accumulation of cholesterol can prevent the activation of pro-inflammatory macrophages and promote the polarization of M2-type macrophages, thereby affecting the inflammatory response of macrophages. For cholesterol metabolism, M2 macrophages also differ from M1 macrophages. M2 polarization signals can downregulate the expression of cholesterol efflux proteins such as ABCA1, reduce cholesterol efflux, relatively increase the intracellular cholesterol level, lead to cholesterol accumulation, and thus inhibit the IFN-γ response of macrophages.^117^ These cholesterols can participate in the formation of cholesterol-rich lipid raft structures, providing a specific microenvironment for the signal transduction and receptor functions of M2-type macrophages, and promoting them to exert functions such as immune regulation. In addition, in the brain tissue, the interaction between cholesterol efflux and uptake is also involved in the regulation of macrophages. Cholesterol in extracellular vesicles (EVs) secreted by repair-associated macrophages can promote the differentiation of oligodendrocyte progenitor cells and myelin regeneration.^118^ Meanwhile, Zheng Guobin and others found through single-cell experiments that GLSP can correct abnormal lipid accumulation (pathological foam formation) in macrophages, promote the expression of ABCA1 and ABCG1, upregulate genes related to lipid breakdown, downregulate genes related to cholesterol metabolism and lipid synthesis, and also increase the expression of anti-inflammatory factors eNOS, TGFβ, Arg1, and IL-10, exhibiting M2-type anti-inflammatory effects^107^ (Fig. 3).

#### Lipid sources and microenvironmental factors drive macrophage fate

Abnormalities in the content and metabolism of substances from different lipid sources within macrophages can affect the metabolic functions and life activities of macrophages. Here, we classify lipids from different sources into long-chain fatty acids and SCFAs, and long-chain fatty acids can be further divided into SFAs and UFAs (Fig. 3).

The accumulation of SFAs activates pro-inflammatory pathways, thereby increasing the number of M1-type macrophages. SFAs (especially palmitic acid, PA) can exacerbate damage in inflammatory diseases by inducing ceramide synthesis, but they can enhance anti-infection ability.^119^ For instance, sEVs rich in SFAs released by hepatocytes under lipotoxic conditions can activate macrophages/Kupffer cells through TLR4, trigger liver inflammation, and then inhibit hepatocyte insulin signaling through paracrine signals.^120^ At the same time, there is a positive causal effect between SFAs and diseases such as RA.^121^ Some UFAs, similar to SFAs, can promote the progression of inflammation in the body through inflammatory products produced by the metabolism of UFAs. Among them, trans fatty acids are related to inflammation. A higher plasma linolelaidic acid content can increase the levels of inflammatory markers and oxidative stress.^122^ In addition, some UFAs can exert anti-inflammatory effects and promote the M2 polarization of macrophages. For example, resolvin E1 derived from n-3 polyunsaturated fatty acid (PUFA) enhances the polarization of macrophages to the M2 type, exerts an inhibitory effect on calcification, and shows a reduction in valvular calcification and a decrease in the area of aortic valve leaflets, thereby inhibiting the progression of aortic valve stenosis.^123^

Compared with long-chain fatty acids, SCFAs have certain anti-inflammatory effects in the human body and can regulate the differentiation of macrophages into an anti-inflammatory phenotype. For example, NaBu inhibits HDAC and enhances the acetylation of the NF-κB p65 subunit, thereby suppressing the inflammatory response of pro-inflammatory macrophages and inducing their conversion to a repair phenotype.^124^ In terms of medicinal use, some drugs can affect SCFAs to achieve certain medicinal effects. For example, metformin can regulate the function of gut microbiota, increase the production of SCFAs, and regulate the differentiation of macrophages into the M2 phenotype.^125^ In addition, similar findings have also been made in the field of traditional Chinese medicine. Gan Wang et al. studied and found that ginsenoside Rg3 can significantly enrich SCFA-producing bacterial genera such as Blautia and Faecalibaculum in the intestine, promote the production of acetic acid and propionic acid, and then activate the type I interferon (IFN-I) response of macrophages through the GPR43-cGAS-STING axis.^126^ At the same time, under physiological conditions, acetic acid is also a necessary condition for the metabolic adaptation and functional maturation of microglia.^127^

In addition to lipids from different sources, the life activities of macrophages in different microenvironments are also affected to a certain extent. In the lipid microenvironment, macrophages are stimulated by lipids, and the expression of low-density lipoprotein receptor-related protein 5 (LRP5) increases and co-localizes with lipids. Under lipid stimulation, LRP5 translocates from the cytoplasm to the cell membrane, participates in the lipid uptake process, and then leads to the accumulation of intracellular CE and induces an inflammatory response.^128^ In the inflammatory microenvironment, the miR-1246 carried by HDL increases and directly targets macrophage DUSP3, downregulating the expression of genes such as DUSP3. This, in turn, increases the expression of IL-6, leading to abnormal HDL function and a weakened anti-inflammatory effect of HDL.^129^ In the TME, EVs secreted by tumors are rich in SFAs (such as palmitic acid). In the TME, abnormal lipid metabolism can lead to the phenotypic conversion of TAMs. For example, SFAs activate the pro-inflammatory phenotype through TLR4, while UFAs promote M2 polarization.^130,131^

### Cooperative regulation of key transcription factors

#### SREBP2

Transcription factor SREBP2 has important regulatory significance in the lipid metabolism pathway of macrophages, mainly regulating the expression of genes involved in cholesterol biosynthesis. Under the inflammatory response, intracellular fatty acid signals can interact to promote the recruitment of macrophages induced by LPS, exacerbating the progression of inflammation. In macrophages, 15-lipoxygenase type B (ALOX15B) increases the levels of the sterol biosynthesis intermediates desmosterol and 27-hydroxycholesterol through the ERK1/2-SREBP2 pathway, thereby enhancing FAO in macrophages. Additionally, SREBP2 inhibits osteoclastogenesis by regulating interferon regulatory factor 7 (IRF7).^106,132^ Immune cells can take up cholesterol through receptor-mediated endocytosis, mainly by taking up LDL via the low-density lipoprotein receptor (LDLR). Among them, LDLR is an important transcriptional target of SREBP2, and its function is related to the activity of SREBP2.^133^ Meanwhile, new experiments have found that Tim-4 is a negative regulator of the antiviral innate immune response in macrophages. It promotes the activation of SREBP2 and cholesterol biosynthesis by interfering with the Insig1-SCAP interaction, thereby inhibiting the type I IFN response and creating favorable conditions for viral infection.^134^ In addition, the MyD88-dependent PRRs signaling pathway activates AKT/mTOR, increases the transcriptional activity of SREBP2, and enhances the synthesis and uptake of cholesterol^135^ (Table 1).

**Table 1:** Co-regulation of key transcription factors

Transcription factors	Transcription target	Functions	Mechanism	Synergistic regulatory mechanism	Ref.
SREBP2	LDLR	Regulate the expression of cholesterol synthase to increase the synthesis and uptake of cholesterol.	Through the ALOX15B-ERK1/2-SREBP2 pathway, it increases intracellular cholesterol content, enhances FAO, exhibits anti-inflammatory polarization, and regulates IRF7 to inhibit osteoclastogenesis.	The PRRs signaling pathway on which MyD88 depends can activate AKT/mTOR, increase the transcriptional activity of SREBP2, and enhance the synthesis and uptake of cholesterol.	^106,132,135^
PPARα	Multiple lipid-lowering enzymes	Promote LD generation and FAO	FAs enter macrophages through MSR1, activate PPARα, promote the generation of LD and FAO. FAO upregulates the expression of BMP7 through the NAD⁺/SIRT1/EZH2 axis, thus promoting bone regeneration.	Magnetic signals regulate macrophage polarization through the PPAR/JAK-STAT signaling pathway and fatty acid metabolism, promote M2 phenotype polarization, and accelerate the migration of M2 macrophages into the bone defect.	^92,136^
LXRβ	Transporter proteins Abca1 and Abcg1	Regulate the excess intracellular cholesterol to be excreted from cells through the cholesterol efflux pathway	LXR promotes cholesterol efflux by transcriptionally activating the genes encoding the transporters Abca1 and Abcg1, exhibiting an anti-inflammatory phenotype.	Activation of LXRβ can significantly inhibit RANKL-induced osteoclast formation and bone resorption function, and downregulate the expression of osteoclast-specific genes TRAP, CTSK, and NFATc1.	^139^
LXRα	NF-κB	Promote the phosphorylation and nuclear translocation of NF-κB and upregulate intracellular cholesterol	The expression of lipogenic enzymes and LXRα in macrophages is upregulated, which enhances the LPS-induced inflammatory response and affects the pro-inflammatory polarization of macrophages.	The upregulation of LXRα expression promotes the phosphorylation and nuclear translocation of NF-κB, affects the pro-inflammatory polarization of macrophages, and at the same time, synergizes with oxLDL to activate RANKL to promote osteoclast differentiation.	^140,141^
STAT1	Promoter of glycolytic genes	Macrophages undergo metabolic reprogramming, with enhanced glycolysis.	CCL7 upregulates the expression of KAT2A to enhance STAT1 succinylation, and STAT1 binds to the promoter of glycolytic genes.	Macrophage metabolic reprogramming, increased glycolysis, and polarization of macrophages to the M1 type. STAT1 can inhibit osteoclast formation by activating the IFN-β signaling pathway.	^150,151^
STAT2	DHCR7	Increase the levels of intracellular cholesterol esters and extracellular 27HC	Regulate macrophage lipid metabolism through the TMEM147/STAT2/DHCR7/27HC axis.	Increased levels of intracellular cholesterol esters and extracellular 27HC, and M2 polarization of macrophages.	^149^
STAT3	NF-κB	Reduce inflammatory response	High expression of MS4A4A in TAM inhibits the NF-κB pathway through the JAK2/STAT3 pathway.	Downregulate the activity of the NLRP3 inflammasome, reduce inflammation, and polarize macrophages towards the M2 phenotype. Meanwhile, STAT3 cooperates with Msh homeobox 1 (MSX1) to drive osteoblast differentiation by promoting the transcription of distal-less homeobox 5 (Dlx5).	^147,148^
STAT6	M2-related genes	Increased molecular expression of the M2 phenotype	Increased FAO levels can activate the STAT6 signaling pathway. The upstream gene JAK2 regulates target genes such as IL-10 and IL-4Ra by activating STAT6, thereby promoting M2 macrophage polarization.	Macrophages polarize to M2, and their anti-inflammatory ability increases. IL-4 and IL-13 induce OPG expression by activating STAT6 in endothelial cells, thereby inhibiting the polarization of osteoclasts.	^144,146^

#### PPARα

PPARα-related regulatory signals also play an important role in lipid metabolism of macrophages. Apoptotic chondrocytes (Acs) can regulate the metabolism and function of macrophages by releasing FAs, promoting the osteogenic differentiation of BMSCs. The released FAs can enter macrophages through MSR1, activate PPARα, and promote the generation of lipid droplets (LD) and FAO. FAO upregulates the expression of BMP7 through the NAD⁺/SIRT1/EZH2 axis, thus promoting bone regeneration.^92^ In addition, Xiaoqing Sun et al. experimentally found that magnetic signals regulate macrophage polarization through the PPAR/JAK-STAT signaling pathway and fatty acid metabolism. Magnetic signals promote the polarization of macrophages towards the M2 phenotype both in vitro and in vivo, and accelerate the migration of M2 macrophages into the traumatic bone defect.^136^ At the same time, some studies have also found that macrophage angiotensin-converting enzyme (ACE) can improve lipid metabolism by increasing peroxisome PPARα, alleviate atherosclerosis, and polarize towards M2^137^ (Table 1).

#### LXR

Excess cholesterol in macrophages is excreted from the cells through the cholesterol efflux pathway regulated by the liver X receptor (LXR). Therefore, LXRs are involved in regulating cholesterol homeostasis and inflammatory responses, and are also crucial for maintaining the survival and immune responses of macrophages.^138^ After LXRβ activation, it will transcriptionally activate the genes encoding the transporters Abca1 and Abcg1, promote cholesterol efflux, and can significantly inhibit RANKL-induced osteoclast formation and bone resorption function. Accordingly, single-cell studies have found that the LXRβ agonist B9 can downregulate the expression of osteoclast-specific genes TRAP, CTSK, and NFATc1.^139^ In addition, in RA, studies have found that the expression of macrophage lipogenic enzymes and LXRα is upregulated, thereby enhancing the lipopolysaccharide (LPS)-induced inflammatory response, promoting the phosphorylation and nuclear translocation of nuclear factor-κB (NF-κB), and affecting macrophage polarization.^140^ Meanwhile, LXRα can synergize with oxLDL to activate RANKL to promote osteoclast differentiation^141^ (Table 1).

#### STAT

In macrophages, the abnormal activation of the Janus kinase/signal transducer and activator (JAK/STAT) pathway can regulate the fatty acid composition (reduce SFAs such as palmitic acid and stearic acid), downregulate lipotoxicity-related genes (such as CD36 and FABP1), and upregulate lipid efflux genes (ABCA1/G1).^142^ TAMs can enhance lipid uptake through the scavenger receptor CD36 on their surface. At the same time, genes related to FAO are highly expressed in TAMs. TAMs utilize FAO for energy supply. Inhibiting FAO will inhibit mitochondrial OXPHOS and ATP production. An increase in the FAO level can activate the STAT6 signaling pathway, promoting the polarization of macrophages towards the M2 phenotype, which can lead to the metastasis of tumor cells.^143^ The upstream gene JAK2 regulates target genes such as IL-10 and IL-4 by activating STAT6, thereby promoting M2 macrophage polarization.^144^ IL-4 and IL-13 induce OPG expression by activating STAT6 in endothelial cells, thereby inhibiting the polarization of osteoclasts.^145,146^ Meanwhile, high expression of MS4A4A in TAM can inhibit NF-κB through the JAK2/STAT3 pathway, downregulate the activity of the NLRP3 inflammasome, and reduce inflammation.^147^ Meanwhile, STAT3 cooperates with Msh homeobox 1 (MSX1) to drive osteoblast differentiation by promoting the transcription of distal-less homeobox 5 (Dlx5).^148^ In addition, Jingjing Huang et al. also discovered the TMEM147/STAT2/DHCR7/27HC axis by which macrophages regulate lipid metabolism. TMEM147 can promote the expression of DHCR7 by activating the transcription factor STAT2, thereby increasing the levels of intracellular cholesterol esters and extracellular 27HC. Subsequently, 27HC promotes the M2 polarization of macrophages.^149^ Unlike many STAT molecules, the chemokine C-C motif ligand 7 (CCL7) enhances the succinylation of STAT1 by upregulating KAT2A expression, which leads to increased binding of STAT1 to the promoter of macrophage glycolytic genes, resulting in metabolic reprogramming of macrophages and their polarization towards M1.^150^ However, STAT1 can inhibit osteoclast formation by activating the IFN-β signaling pathway.^151^ In summary, most members of the STAT family activate the expression of downstream M2-related factors through the (JAK/STAT) pathway, thereby polarizing macrophages towards the M2 type, while STAT1 can polarize macrophages towards the M1 type by regulating their metabolic reprogramming (Table 1).

## The dual roles of macrophage lipid metabolism in inflammatory bone diseases and bone remodeling

### Macrophage abnormalities driven by lipid metabolism disorders and the occurrence of inflammatory bone diseases

When the immune protection/regulation microenvironment of the remodeling unit is imbalanced (such as chronic inflammation, excessive hypoxia, and disorder of the cytokine network), the morphology and function of macrophages will undergo abnormal changes, which in turn can cause bone metabolic diseases.^152^ Inflammatory bone diseases such as rheumatoid arthritis (RA) and osteoporosis are closely related to a series of related changes triggered by macrophage lipid metabolism disorders in terms of their pathogenesis. In inflammatory bone diseases, lipid metabolism disorders caused by metabolic disorders of lipid components such as fatty acids and cholesterol in macrophages can affect the polarization of macrophages into M1 macrophages, exacerbating the body’s inflammatory response and thus aggravating the course of related diseases.^153^ At the same time, lipid metabolism disorders lead to changes in the cytokines secreted by macrophages. For example, pro-inflammatory factors such as TNF-α and IL-1 increase. These factors stimulate the differentiation of osteoclast precursors into mature osteoclasts, enhance the bone resorption ability of osteoclasts, and at the same time inhibit the activity of osteoblasts and reduce bone formation.^154,155^

#### Rheumatoid arthritis (RA)

**Fig. 4:**
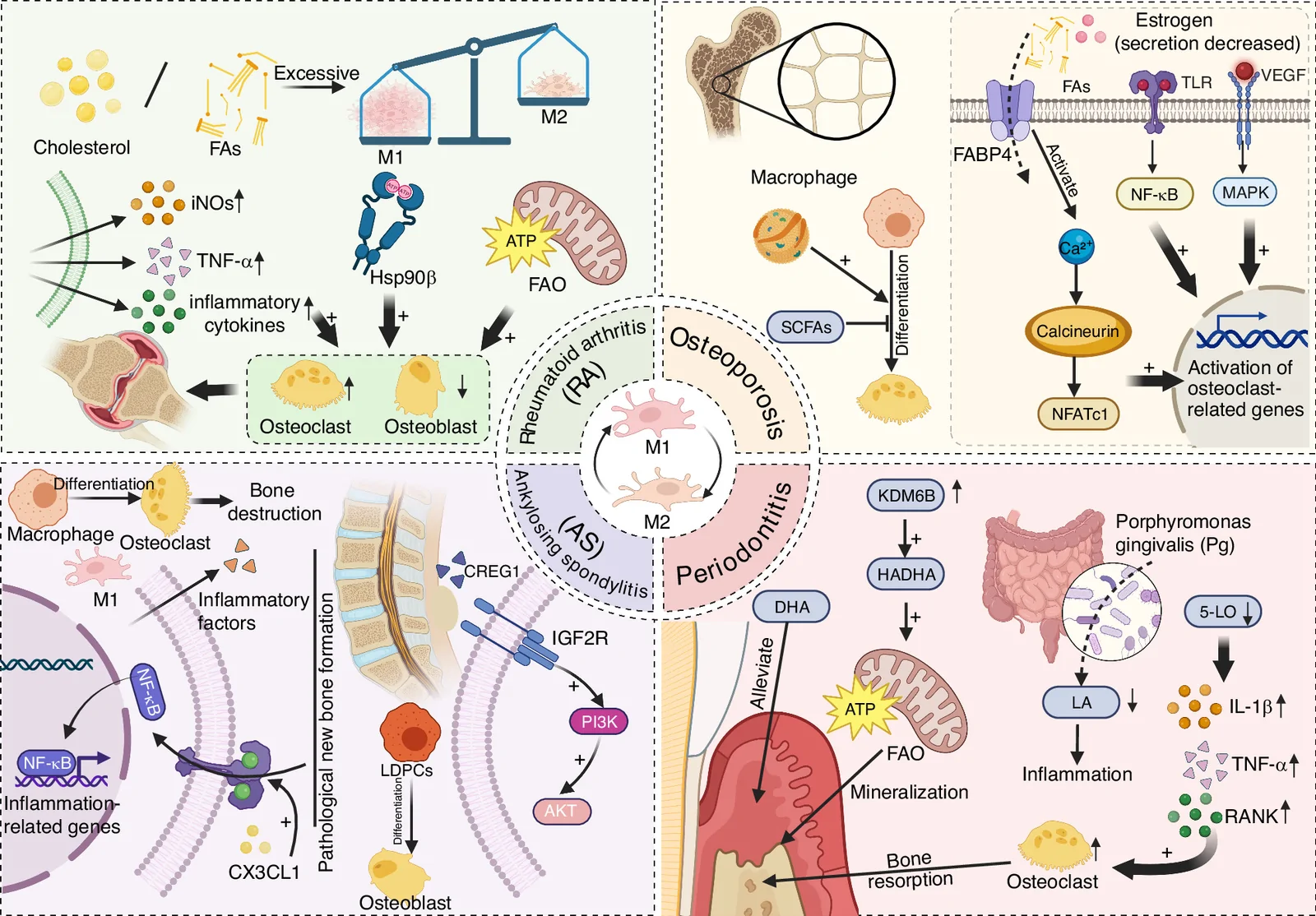
Mechanisms of macrophage lipid metabolism in inflammatory bone diseases. This diagram systematically elaborates on the multi-dimensional molecular mechanisms by which they induce bone metabolism imbalance in diseases such as rheumatoid arthritis, osteoporosis, periodontitis, and ankylosing spondylitis through regulating inflammatory responses, lipid metabolism, and osteoblast/osteoclast activity

RA is a representative chronic autoimmune bone disease, and genes such as HLA-DRB1 have been proven to be related to its pathogenesis.^156^ When lipid metabolism is imbalanced, excessive fatty acids or cholesterol can promote the polarization of macrophages into M1 type, leading to an increase in the number of M1 macrophages. For example, the impairment of the ATP-binding cassette G1 (ABCG1) membrane transporter protein weakens the cholesterol efflux capacity (CEC), exacerbating inflammation.^157^ The large amount of pro-inflammatory cytokines secreted by M1 macrophages can not only exacerbate the inflammatory response but also interfere with the normal functions of osteoclasts and osteoblasts, thereby disrupting bone homeostasis. Furthermore, recent studies have found that FABP4 secreted by M1-polarized macrophages can promote angiogenesis and cellular activities by activating VEGFα expression and the MAPK pathway, induce the production of inflammatory cytokines through the NF-κB pathway, and promote the degradation of acid mucopolysaccharides in chondrocytes, disrupting chondrocyte homeostasis and exacerbating the patient’s condition^158^ (Fig. 4).

In the bone tissue microenvironment, macrophages have close interactions with osteoclasts, osteoblasts, etc., jointly maintaining bone homeostasis. However, lipid metabolism imbalance can disrupt the normal communication between macrophages and these cells, leading to abnormal life activities of related cells and exacerbating the disease course. Upregulated FAO promotes monocyte recruitment and osteoclast-like cell differentiation, interacts with fibroblast-like synoviocytes (FLS), and contributes to the inflammatory phenotype of RA-FLS.^159^ Besides, Heat shock protein 90β (Hsp90β), as an important molecular chaperone, also plays a key role in osteoclastogenesis. c-Jun can lead to the pathological upregulation of Hsp90β, and Hsp90β promotes osteoclastogenesis by dual activation of cholesterol synthesis and NF-κB signaling.^160^ SREBP2, as a key regulator of cholesterol synthesis, can regulate the accumulation of cholesterol in cells. Moreover, the interaction between cholesterol and estrogen-related receptor α (ERRα) can promote the formation of osteoclasts. Based on this, SREBP2 and ERRα have become important therapeutic targets in women with RA after menopause.^161^

#### Osteoporosis

Imbalance in bone remodeling is the core of Osteoporosis. For example, postmenopausal estrogen deficiency, oxidative stress caused by aging, chronic inflammation, etc., can activate pathways such as NF-κB and MAPK, promoting osteoclast differentiation and inhibiting osteoblast function.^162^ Postmenopausal osteoporosis (PMOP) caused by estrogen deficiency is mainly manifested as bone loss and an increased risk of fractures. Among them, fatty acid-binding protein 4 (FABP4), as a key protein involved in lipid metabolism and inflammation, can promote osteoclast differentiation and exacerbate PMOP by activating the Ca^2+^-calcineurin-NFATc1 pathway^163^ (Fig. 4).

In osteoporosis, oxidized low-density lipoprotein (ox-LDL) interferes with the normal functions of macrophages, causing an imbalance in the cytokines they secrete, promoting the generation and activation of osteoclasts, inhibiting the functions of osteoblasts, which results in reduced bone formation and enhanced bone resorption. Abnormal metabolism of cholesterol related to HDL can also exacerbate the disease process. Macrophages can take up cholesterol through a variety of scavenger receptors and excrete cholesterol through the ABCG5/8 and TICE pathways. Reducing cholesterol levels by inhibiting cholesterol biosynthesis and uptake, or enhancing cholesterol utilization and excretion, can slow down osteoporosis.^164^ The long-chain and very-long-chain fatty acid transporter FATP2 can affect osteoclast differentiation by regulating fatty acid uptake and energy metabolism.^165^ In contrast, UFAs can directly modulate osteoclast and osteoblast differentiation through the FFAR4/β-arrestin 2 pathway, thereby contributing to bone-homeostatic regulation.^166^ To this end, recent studies have pointed out that gut microbiota-related metabolites participate in the regulation of bone homeostasis through the “gut-bone axis” and influence the progression of osteoporosis through SCFAs, bile acids, choline metabolites, as well as estrogen and dietary polyphenols. For example, supplementation with propionic acid and butyric acid can significantly improve bone loss in ovariectomized mice^167^; ω-3 fatty acids inhibit osteoporosis by promoting calcium absorption, inhibiting pro-inflammatory factors (such as IL-6, TNF-α), reducing osteoclast activation and bone resorption, and directly stimulating osteoblast activity.^168^

#### Ankylosing spondylitis (AS)

Ankylosing spondylitis (AS) is a chronic inflammatory disease that involves the axial skeleton and sacroiliac joints. Xinzhe Feng et al. found that in AS, CX3CL1 promotes M1 macrophage polarization by activating the NF-κB signaling pathway, increases the expression of inflammatory factors, and promotes osteoclast differentiation, resulting in bone destruction.^169^ In addition, AS is also characterized by pathological new bone formation that can lead to disability. In the areas of new bone formation and adjacent to osteogenic precursor cells, there is an increase in triggering receptor expressed on myeloid cells 2^+^ (TREM2^+^) macrophages. The CREG1-IGF2R-PI3K-AKT signaling pathway mediates the osteogenic differentiation of ligament-derived progenitor cells (LDPCs), resulting in pathological new bone formation.^170^ Research has also found that AS is associated with cardiovascular diseases. Patients with AS have lower levels of high-density lipoprotein cholesterol and elevated C-reactive protein (CRP).^171^ After treatment with statins, low-density lipoprotein cholesterol can be reduced, and CRP-related inflammation can be alleviated.^172^ In other aspects, the regulation of lipid metabolism in AS still needs to be studied. Single-cell RNA sequencing (scRNA-seq) shows that Gnas expression is increased in the macrophage cluster involved in cholesterol metabolism in the atherosclerotic aorta, and the activity of Gsα (G protein stimulatory α subunit) protein is also upregulated. Moreover, oxidized low-density lipoprotein (Ox-LDL) can translocate Gsα in macrophage lipid rafts in the short term and promote Gnas transcription in the long term through oxidative stress, leading to increased lipid uptake and foam cell formation^173^ (Fig. 4).

The “gut-bone axis” also has a certain regulatory effect on AS. SCFAs and medium-chain fatty acids can regulate immune cells to relieve joint inflammation and bone loss.^174^ Meanwhile, the study found that patients with AS have gut microbiota dysbiosis (enrichment of adherent and invasive bacteria), upregulation of zonulin leading to disruption of the intestinal epithelial and vascular barriers, increased levels of LPS, intestinal fatty acid-binding protein (iFABP), and zonulin in the serum. Meanwhile, single-cell studies have found that circulating monocytes exhibit an anergic phenotype.^175^

#### Periodontitis

Periodontitis is a common chronic inflammatory disease triggered by bacteria, which can lead to bone resorption, tooth loss, and systemic inflammation. Long-chain ω-3 fatty acids, such as docosahexaenoic acid (DHA), can alleviate the symptoms of periodontitis. Supplementation with DHA reduces the average periodontal pocket depth and gingival index.^176^ Research has found an association between metabolic syndrome (MetS) and an increased risk of periodontitis. A high-fat diet significantly exacerbates LPS-induced alveolar bone loss, osteoclastogenesis, and inflammatory infiltration. Palmitic acid can significantly enhance the pro-inflammatory gene expression of macrophages induced by LPS.^177^ At the same time, it was found that obesity is related to abnormal lipid metabolism and impaired bone homeostasis. In an environment with a high level of high-fat palmitic acid (PA), the inflammatory response of osteoclasts to Porphyromonas gingivalis is enhanced, which in turn affects alveolar bone loss.^178^ In terms of fatty acid oxidative metabolism, promoting the expression of lysine-specific demethylase 6B (KDM6B) can regulate HADHA by mediating histone demethylation and lactylation, thereby upregulating FAO and further promoting mineralization.^179^ In addition, diabetes is also an important risk factor for periodontitis.^180^ In diabetic patients, high blood sugar can disrupt the bone immune environment, causing an imbalance in macrophage types—more M1 and fewer M2—which leads to excess pro-inflammatory factors and insufficient anti-inflammatory factors, ultimately delaying fracture healing.^181^ Activating the anti-inflammatory phenotype can promote fracture healing.^182^ Meanwhile, the main differentially expressed molecule, fatty acid synthase (FASN) in diabetic exosomes, leads to ectopic fatty acid synthesis in PDLCs and activates the cleavage of gasdermin D, thereby exacerbating bone loss.^183^ Metformin (Met) alleviates the inflammatory response in diabetic periodontitis by activating AMPK and inhibiting the NF-κB signaling pathway. It can significantly reduce inflammatory cell infiltration, collagen degradation, and osteoclast formation, thereby mitigating alveolar bone loss.^184^ In addition, the adiponectin (APN) agonist AdipoAI can attenuate lipopolysaccharide (LPS)-induced activation of NF-κB P65 and expression of CCL2, and reduce alveolar bone resorption and gingival inflammation^185^ (Fig. 4).

The microbiota in the microenvironment of periodontitis plays an important regulatory role in the course of periodontitis and metabolism. The salivary microbiota of periodontitis can reduce the level of UFAs, promote the metabolism of arachidonic acid, regulate macrophage polarization, and aggravate the condition of periodontitis.^186^ The periodontal pathogen Porphyromonas gingivalis (Pg) can inhibit the linoleic acid (LA) pathway in the gut microbiota in a gut microbiota-dependent manner, exacerbating the inflammatory response.^187^ In addition, 5-lipoxygenase (5-LO) plays a key role in the inflammatory response triggered by bacteria. Apical periodontitis (AP) is an immune disorder disease caused by the imbalance between bacterial invasion and host defense response. In AP, the low expression of 5-LO weakens the host innate immune system, enhances the expression of inflammation- and osteoclastogenesis-related factors such as IL-1β, TNF-α, RANK, and RANKL, while the level of osteoprotegerin decreases, macrophage polarization is abnormal, osteoclast differentiation increases, and bone resorption is aggravated.^188^

### Anti-inflammatory lipid-mediated macrophage reprogramming and regulation of bone repair

**Fig. 5:**
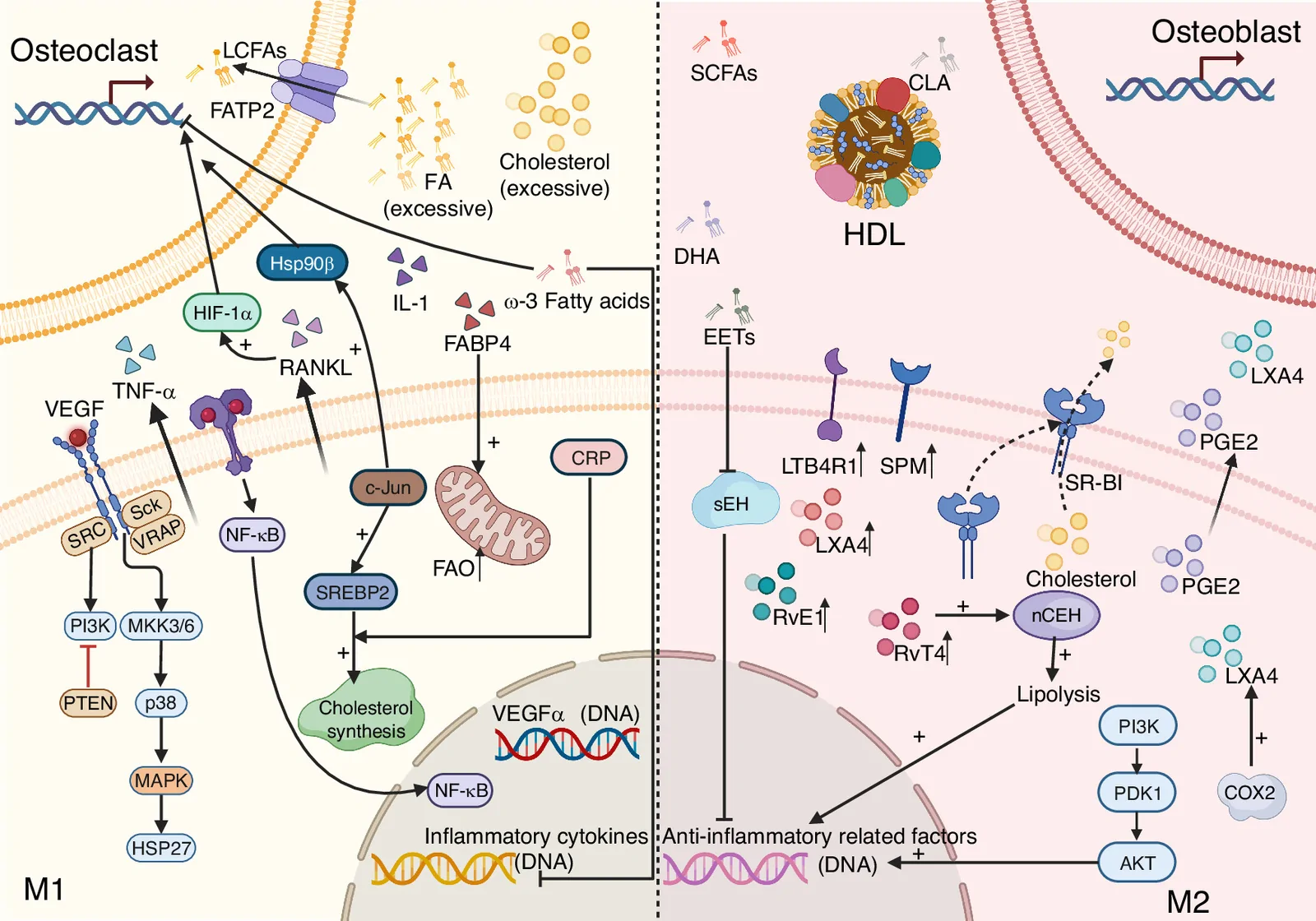
Bidirectional regulation of lipid metabolism and signaling pathways in osteoclasts and osteoblasts. Excessive fatty acids, cholesterol, etc., activate NF-κB through FATP2 and RANKL to promote cholesterol synthesis, while FABP4 mediates FAO. Short-chain fatty acids (SCFAs), DHA, etc., activate the PI3K/AKT pathway through HDL, SR-BI, etc., promote lipolysis and transcription of anti-inflammatory factors, inhibit inflammation, and contribute to osteoblast-related bone formation

Lipids can affect the polarization and life activities of macrophages by regulating their metabolic reprogramming. Anti-inflammatory lipid molecules, on the other hand, are a class of molecules that regulate the lipid composition of macrophages, influence the macrophage signal transduction pathway, and thus promote the formation of an anti-inflammatory phenotype. There are many types of anti-inflammatory lipid molecules, including various anti-inflammatory lipid mediators derived from PUFAs represented by Resolvins, structural lipid components such as HDL, and gut-derived or diet-related anti-inflammatory lipid metabolites such as SCFAs.^189–191^ Most of them have been experimentally verified to play an important role in macrophage-mediated bone remodeling (Fig. 5).

#### Anti-inflammatory lipid mediators derived from polyunsaturated fatty acids (SPMs class)

Anti-inflammatory lipid mediators derived from PUFAs are mainly generated by the enzymatic metabolism of ω-3 or ω-6 fatty acids. They have the function of “pro-resolution of inflammation,” are highly active, and have clear action targets. Inflammation resolution is an actively regulated process coordinated by specialized pro-resolving mediators (SPMs), which can clear cellular debris and immune cells in tissues and restore homeostasis.^192^ As endogenous bioactive lipids, SPMs, including lipoxins, resolvins, protectins, and maresins, etc., have insufficient production, which is associated with various inflammatory diseases.

Resolvins have significant potential in regulating the phenotype and function of immune cells, promoting the resolution of inflammation, improving insulin resistance, wound healing, and vascular homeostasis. At the same time, the Resolvins family has many subdivisions, including RvE1, RvD1, RvT4, etc., and their functions have certain differences. Resolvin E1 (RvE1) inhibits neutrophil chemotaxis through ChemR23, and resolvin D1 (RvD1) enhances macrophage phagocytic function through ALX/FPR2, thereby reducing the inflammatory response in the periosseous microenvironment and promoting the healing of inflammatory bone injuries.^193^ RvT4 promotes the decomposition of neutral lipids by activating neutral cholesterol ester hydrolase (nCEH), and at the same time promotes the translocation of scavenger receptor B1 (SR-BI) to the cell membrane, enhances cholesterol efflux from macrophages, reprograms macrophages to polarize towards the M2 type, and exhibits an anti-inflammatory phenotype.^111^

As an important component of SPMs, Protectins can regulate the polarization of macrophages and the secretion of inflammatory factors, exerting an anti-inflammatory effect. Neuroprotectin D1 (NPD1) induces an increase in the intracellular calcium ion concentration in macrophages through the Gi-protein-coupled pathway, and depends on calcium ion signals and the PI3K/AKT and ERK pathways to enhance the phagocytic ability of macrophages and promote the polarization of macrophages towards an anti-inflammatory phenotype.^194^ In addition, new research has found that PD_n−3_ DPA derived from n-3 DPA is a new type of pro-resolution mediator of inflammation. PD_n−3_ DPA can regulate the differentiation of monocytes into macrophages with an anti-inflammatory phenotype, enhance their efferocytosis and phagocytic functions, and promote the resolution of inflammation.^195^ Moreover, ALN in DPA can reduce bone destruction by inhibiting the activity of osteoclasts and at the same time induce the continuous deposition of minerals.^196^ Maresins play an important immunological role in regulating macrophage infiltration and the polarization of the anti-inflammatory phenotype in both humans and animals. Maresin-1 (MaR1) can reduce the levels of various pro-inflammatory cytokines (such as IL-1α, IL-1β, TNFα, IFNγ) and chemokines (such as CCL-2, CCL-5) in the spinal cord, decrease the infiltration of immune cells like macrophages, reduce the number of pathogenic Th1 cells, and promote the polarization of macrophages towards an anti-inflammatory phenotype (such as an increase in CD206^+^ macrophages).^197^ C W Wang et al. found that in the healing of tooth extraction wounds and alveolar bone regeneration, local application of MaR1 can accelerate the healing of tooth extraction wounds by promoting epithelial regeneration, accelerating alveolar bone filling, and regulating the polarization of macrophages towards an anti-inflammatory phenotype.^198^

#### Anti-inflammatory components in structured lipids or metabolic lipids

Anti-inflammatory components in structural lipids or metabolic lipids are intermediate products or storage-transport molecules in the lipid metabolism process and have anti-inflammatory effects under specific conditions. Structural lipids mainly include high-density lipoprotein (HDL) and its apolipoprotein miRNAs (such as miR-223), while there are a variety of metabolic lipids, including oxidized bile acids (such as TUDCA), 2-arachidonoylglycerol (2-AG), etc.

High-density lipoprotein (HDL) can regulate the intracellular cholesterol content and macrophage polarization in the body. Among them, HDL, as a reverse cholesterol transporter, can transfer cholesterol from macrophages to the liver for processing and excretion into bile. At the same time, HDL and its contained apolipoprotein AI (apoAI), paraoxonase 1 (PON1), and other proteins can effectively prevent cellular oxidative stress and abnormal modification of LDL, and inhibit the inflammatory signal transduction of macrophages and other cells.^199^ In hepatocytes, the transcription factor ATF3 has an anti-inflammatory function. ATF3 can enhance the uptake of high-density lipoprotein (HDL) by interacting with p53, inhibit cholesterol 12α-hydroxylase (CYP8B1) by interacting with hepatocyte nuclear factor 4α, and promote reverse cholesterol transport in macrophages.^200^ In addition, HDL can also reduce the activity of osteoclasts and promote their apoptosis, which plays an important role in bone diseases caused by hyperlipidemia and the vascular system.^201^

Bile acids can promote the polarization of macrophages towards an anti-inflammatory phenotype and inhibit the release of inflammatory molecules in the body. As an anti-inflammatory signaling mediator, bile acids can activate their nuclear receptor and induce the polarization of M2-like macrophages. Supplementing bile acid in daily life or preventing excessive loss of bile acid may have an anti-inflammatory effect.^202^ Ursodeoxycholic acid (UDCA) binds to the bile acid receptor FXR, inhibits the activation of nuclear factor κB (NF-κB) in macrophages, induces M2 macrophage polarization, and reduces the production of inflammatory cytokines.^203^ In addition, hyodeoxycholic acid (HDCA) can inhibit osteoclast maturation and exert a bone-protective effect in vivo by activating the G protein-coupled bile acid receptor (TGR5).^204^ Meanwhile, bile acids can significantly promote osteoblast differentiation by upregulating Runx2 and enhancing the extracellular signal-regulated kinase (ERK) and β-catenin signaling pathways.^205^

Among endocannabinoids, 2-arachidonoylglycerol (2-AG), as an anti-inflammatory component in metabolic lipids, can promote the polarization of M2 macrophages. WWL70 can inhibit the 2-AG hydrolase α-β hydrolase domain 6 (ABHD6), increase the content of 2-AG, exert anti-inflammatory effects, and relieve inflammatory pain.^206^ Diacylglycerol lipase-β (DAGLβ) serves as a biosynthetic enzyme for 2-arachidonoylglycerol (2-AG). The disruption of DAGLβ leads to the activation of the LKB1-AMPK signaling pathway, resulting in the reprogramming of the phosphoproteome and bioenergetics. The reduction of 2-AG triggers inflammation.^207^ Moreover, current studies have found that the differentiation process of monocytes into osteoclasts is accompanied by a decrease in 2-AG levels and an increase in AEA levels.^208^

#### Anti-inflammatory lipid metabolites derived from the intestine or diet

Most of the anti-inflammatory lipid metabolites of intestinal origin or related to diet are nutritional metabolites or microbial fermentation derivatives. They have the potential for immunomodulation and tissue repair, mainly including SCFAs and UFAs, etc.

Intestinal microorganisms can regulate the content of intestinal SCFAs (such as butyric acid and propionic acid), affect the absorption of SCFAs, thereby influencing the progression of inflammation and bone homeostasis. Meanwhile, SCFAs can also affect intestinal microorganisms. This forms an intestinal-bone axis regulatory system. A growing number of studies have shown that the intestinal flora and their related metabolites play a key role in maintaining bone health and the progression of bone diseases such as OA and osteoporosis. Studies have found that gold nanoparticles (GNPs) and melatonin (MLT) can enhance the diversity of intestinal flora, the relative abundance of various probiotics (such as Allobaculum and Paraclostridium), as well as the metabolic function of intestinal flora, increase the production of SCFAs, reduce trimethylamine N-oxide-related metabolites, regulate the balance of M1/M2 macrophages, decrease the levels of pro-inflammatory cytokines such as TNF-α and IL-12 in serum, increase the levels of anti-inflammatory cytokines such as IL-10 in serum, inhibit osteoclast activity, and reduce the level of bone resorption marker CTX-1.^209,210^ Polyvinyl butyrate nanoparticles (BuNP) can repair the intestinal epithelial barrier, regulate the balance of intestinal flora, inhibit systemic inflammation, thereby restoring the activity balance between osteoblasts and osteoclasts and alleviating bone loss.^211^ Furthermore, studies have confirmed that SCFAs (butyric acid) absorbed in the intestine can inhibit osteoclast differentiation, alter the KEGG pathway of osteoclast differentiation and the expression of HDAC, and reduce bone destruction.^212,213^

Numerous research data on ω-3 fatty acids and their precursors (EPA, DHA) have also found that Omega-3 fatty acids have anti-inflammatory activity and are beneficial for autoimmune diseases. It has been found through multiple single-cell research data that ω-3 fatty acids and DHA can reduce the risk of RA and systemic lupus erythematosus, and can improve disease activity and inflammatory markers.^214^ However, the mechanism of action of Omega-3 is not fully understood. In the inflammatory environment induced by TNF-α, high-dose eicosapentaenoic acid (EPA) (100 μmol/L) can reverse the inhibitory effect of TNF-α on the osteogenic differentiation of mBMSCs, increase calcium deposition and ALP activity, and upregulate the expression of osteogenesis-related genes and proteins. At the same time, it inhibits the activation of the NF-κB signaling pathway in mBMSCs stimulated by TNF-α, reduces the expression of p-IKK and p-p65. In addition, EPA can also reduce the number of osteoclasts and inhibit the expression of related genes and proteins.^215^

Cannabidiol (CBD) and lipid metabolites influenced by CBD are of great significance in the regulation of inflammation. They can promote the anti-inflammatory phenotypic polarization of macrophages and inhibit the release of inflammatory factors. Cannabidiol (CBD) can regulate the imbalance of gut microbiota in CIA rats and significantly upregulate the levels of L-carnitine and butyric acid in serum. L-carnitine can inhibit the inflammatory responses of neutrophils, macrophages, and RA-FLSs, reduce synovial inflammatory infiltration and inflammatory bone injury, promote synovial cell proliferation, decrease the levels of TNF-α and IL-6 in plasma, reduce the proportion of M1 macrophages in the spleen, and thus improve collagen-induced arthritis (CIA).^216^

In addition to the above common dietary-related fatty acids, a variety of lipid molecules have been shown to have anti-inflammatory effects. For example, conjugated linoleic acid (CLA) induces macrophages to produce IL-35 through the Gαq/11-mediated STAT1/4 pathway, exerting an anti-inflammatory effect.^217^ At the same time, CLA can promote the production of osteoblasts and has a certain potential regulatory effect on osteoclasts.^218^ Ginkgolide (Bil) can inhibit the phosphorylation and nuclear translocation of p65 in the NF-κB signaling pathway after RANKL stimulation, promote the expression of SIRT3, inhibit the NF-κB signaling pathway through the activation of the SIRT3/SOD2/ROS axis, inhibit osteoclast differentiation, and promote the polarization of macrophages to M2;^219^ 2-Aminoethoxydiphenyl borate (2-APB) can dose-dependently reduce the expression of the M1-related gene iNOS, increase the expression of the M2-related gene Arg-1, and promote the transformation of macrophages from the pro-inflammatory M1 phenotype to the anti-inflammatory M2 phenotype.^220^ However, 2-APB can inhibit the proliferation of osteoblasts.^221^ In addition, new research has explored the traditional medicine Ershiwuwei Lvxue Pills (ELP). It was found that ELP can change the abundance of serum metabolites, affect multiple metabolic pathways such as arginine biosynthesis and arachidonic acid metabolism, reduce the level of arachidonic acid, decrease the expression of cytokines such as M-CSF, GM-CSF and PGE2, regulate bone metabolism markers, and inhibit the OPG/RANKL/NF-κB signaling pathway, thereby reducing the production of MMPs and osteoclasts.^222^

## Molecular mechanisms of lipid-driven pathways

### The role of lipid signaling in macrophage-osteoclast differentiation

**Fig. 6:**
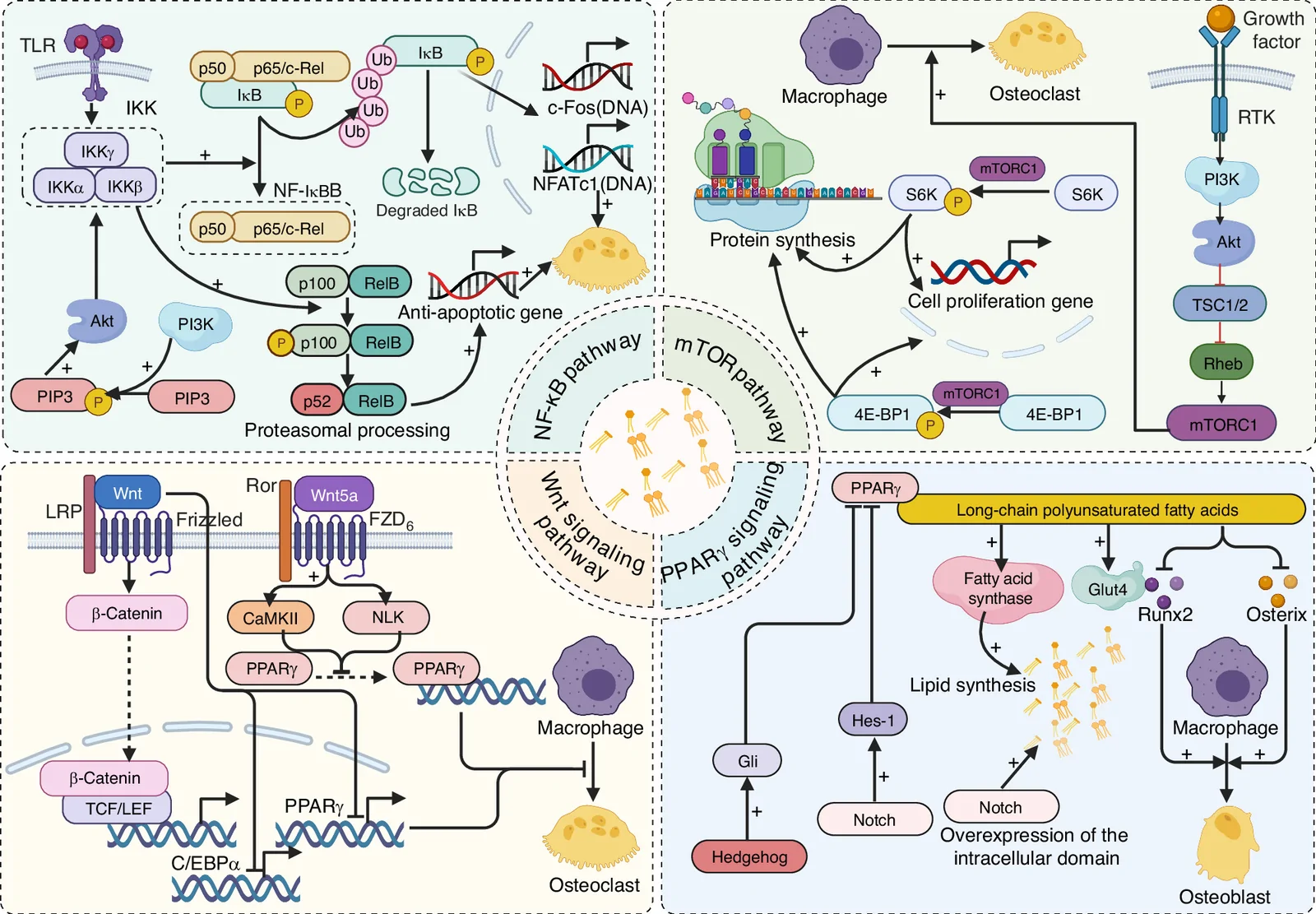
Molecular mechanisms of lipid-driven pathways. NF-κB pathway: TLR signaling activates IKK, which phosphorylates IκB and promotes its degradation, thereby releasing NF-κB and inducing c-Fos and NFATc1 expression to support osteoclastogenesis; mTOR pathway: growth factors activate mTORC1 through RTK/PI3K/Akt, promote protein synthesis and cell proliferation, and drive the differentiation of macrophages into osteoclasts; Wnt pathway: canonical Wnt signaling regulates transcription through β-catenin/TCF-LEF complexes, whereas non-canonical Wnt5a signaling modulates PPARγ through CaMKII-dependent mechanisms; PPARγ pathway: long‑chain polyunsaturated fatty acids activate PPARγ, regulate lipid synthesis, inhibit osteogenesis‑related Runx2 and Osterix, and thereby participate in the regulation of macrophages and osteoclasts

Osteoclasts are mainly formed by the fusion of circulating monocytes and are responsible for degrading bone tissue.^223^ Macrophages, as key precursors, play an important role in the regulation of bone homeostasis. Research shows that lipid signals have a core regulatory role in the process of osteoclast differentiation. During osteoclast differentiation, genes involved in OXPHOS and mitochondrial FAO are significantly upregulated. The oxidation rates of oleic acid and glutamine and the mitochondrial respiration rate in mature osteoclasts are all significantly increased. This suggests that enhanced fatty acid metabolism helps to promote the differentiation and maturation of osteoclasts.^224^ Among them, a variety of transcription factors and signaling molecules are involved in the regulation of macrophage-osteoclast differentiation (Fig. 6).

#### NF-κB pathway

Lipid signals can increase the differentiation of macrophages into osteoclasts by activating the NF-κB pathway. Some lipid signaling molecules, such as the products of phosphatidylinositol-3-kinase (PI3K), can activate the downstream protein kinase B (Akt). Akt can activate IκB kinase (IKK), which phosphorylates IκB and promotes its ubiquitination and degradation, thereby releasing NF-κB.^225^ NF-κB is a key transcription factor for osteoclastogenesis. It can induce the expression of osteoclast-specific transcription factors such as c-Fos and NFATc1. c-Fos and NFATc1 can regulate the expression of osteoclast-related genes such as cathepsin K and tartrate-resistant acid phosphatase (TRAP), thus promoting the formation of osteoclasts and their bone resorption function.^226,227^ In addition, the NF-κB pathway activated by lipid signals can also upregulate the expression of some anti-apoptotic genes, such as members of the Bcl-2 family. This inhibits the apoptosis of osteoclasts, prolongs the lifespan of osteoclasts, and enhances their bone resorption ability.

#### mTOR pathway

Lipid signals can activate the mTOR pathway through multiple pathways and regulate macrophage-osteoclast differentiation. The PI3K-Akt signaling pathway can not only activate NF-κB but also mTOR. Akt can directly phosphorylate the regulatory-associated protein of mTOR (raptor), thereby activating mTOR complex 1 (mTORC1).^228^ In addition, after some growth factors and cytokines bind to their receptors, they can also activate mTOR through the lipid signal-mediated pathway. mTOR plays an important regulatory role in osteoclastogenesis. Among them, mTORC1 can phosphorylate downstream ribosomal protein S6 kinase (S6K) and eukaryotic initiation factor 4E-binding protein 1 (4E-BP1), promoting protein synthesis and cell proliferation.^229,230^ In osteoclast precursor cells, the activation of mTOR contributes to cell proliferation and differentiation into osteoclasts.

#### Wnt signaling pathway

The Wnt signaling pathway is divided into the canonical and non-canonical pathways. It can regulate the differentiation of macrophages into osteoclasts, but it plays opposite roles in bone cancers. In the canonical Wnt signaling pathway, Wnt proteins can stabilize β-catenin, inhibit the transcriptional activities of PPARγ and C/EBPα, suppress adipogenesis, and inhibit osteoclast differentiation.^231^ In the non-canonical pathway, Wnt5a inhibits the binding of PPARγ to DNA and osteoclast differentiation by activating CaMKII and NLK.^232^ But interestingly, in multiple myeloma (MM), the high-risk factor PNPO, as an oxidase, can oxidize disheveled protein 3 (DVL3), leading to the protection of β-catenin from degradation and its entry into the nucleus, activating the Wnt/β-catenin pathway to induce the differentiation of pre-osteoclasts (RAW264.7) into mature osteoclasts and promoting cancer progression.^233^

#### PPARγ signaling pathway

In multiple pathways, PPARγ, as the “master regulator” of adipogenesis, plays an important role in macrophage-osteoclast differentiation and interacts with various signaling molecules to jointly regulate osteoclast differentiation. PPARγ can be activated by binding to ligands (such as long-chain LCPUFAs), induce the expression of adipocyte-specific genes (e.g., fatty acid synthase, Glut4), and simultaneously inhibit the osteogenic transcription factors Runx2 and Sp7 (Osterix), thereby inhibiting the differentiation of MSCs into osteoblasts.^234,235^ The Hedgehog signal interacts with PPARγ, inhibits PPARγ through the Gli transcription factor, promotes osteogenesis, and inhibits adipogenesis^236^; The Notch signal Jagged1 inhibits PPARγ through Hes-1, but overexpression of the Notch1 intracellular domain can promote adipogenic differentiation, and the mechanism is complex.^237^ In addition to signaling molecules, the C/EBPs family, including C/EBPα, β, and δ, can co-activate PPARγ, regulate the cell cycle, and promote adipocyte differentiation.^238^ In addition, BMP signaling influences mesenchymal lineage commitment. BMP-2, BMP-4, BMP-6, BMP-7, and BMP-9 can induce both osteogenic and adipogenic differentiation of mesenchymal progenitor cells, with lineage outcome shaped by the relative activities of Runx2 and PPARγ2.^239^

### Integration of lipid metabolism and cytokine signaling: implications for osteoimmunology

Intracellular lipid metabolism not only provides a certain amount of energy for the life activities of cells, but its metabolites also have a certain regulatory effect on human immunity and can affect the synthesis and secretion of some cytokines in immune cells. For example, arachidonic acid (AA) is metabolized to produce prostaglandin E2 (PGE2) and other eicosanoids.^240^ It can promote the expression of osteoclastogenesis-related cytokines such as receptor activator of RANKL, and at the same time inhibit the expression of OPG, thus indirectly regulating bone metabolism.^241^ In addition, LCPUFAs and their metabolites are considered key factors in maintaining bone and joint health. n-6 PUFAs inhibit osteoblast differentiation and promote osteoclast differentiation by increasing the expression of peroxisome proliferator-activated receptor γ (PPARγ) and promoting adipogenesis, while n-3 PUFAs promote osteoblast formation by downregulating PPARγ and enhancing osteoblast activity.^242–244^

OPG is a soluble decoy receptor for RANKL. It can effectively inhibit the interaction between RANKL and its receptor, receptor activator of nuclear factor-κB (RANK), thereby negatively regulating the differentiation and activation of osteoclasts and promoting the proliferation and differentiation of osteoblasts, thus preventing osteoporosis.^245,246^ After menopause, due to a decrease in estrogen secretion in elderly women, the secretion and function of Osteoprotegerin (OPG) are inhibited, often resulting in osteoporosis.^247^ RvE1, by regulating the RANKL/OPG balance in osteoblasts, inhibits the NF-κB and PI3K-AKT signaling pathways, reduces osteoclastogenesis, and thus protects bone tissue under inflammatory conditions.^248^ In addition, parathyroid hormone (PTH) can also regulate the production of RANKL / OPG through beta-arrestin to control the formation of osteoclasts.^249^

Compared with the effects of lipid metabolism on cytokine production, lipid-binding proteins can also reshape inflammatory and metabolic signaling. In adipocytes, extracellular FABP4 promotes lipolysis and inflammation through p38/HSL and p38/NF-κB signaling, thereby altering lipid handling and increasing inflammatory cytokine expression.^250^ The upregulation of FABP4 increases the transport of fatty acids such as palmitic acid, activates the NLRP3 inflammasome, promotes the release of IL-1β/IL-18 through the caspase-1/GSDMD pathway, and polarizes macrophages to the M1 phenotype.^251^ Meanwhile, during osteoclastogenesis, cytokines such as M-CSF and RANKL can regulate the lipid uptake of osteoclast precursor cells to meet the energy and substances required for their differentiation and function.^252,253^ In addition, the cytokine signaling pathway can also affect the activity and expression of enzymes related to lipid synthesis and metabolism. For example, inhibition of interleukin‑17‑related signaling can alleviate inflammatory responses and osteoclast differentiation.^254^ In addition, the study also found that FNDC5/irisin, as a cytokine released by muscles after exercise, activates osteocytes in females to promote bone resorption to provide calcium required for reproduction, increasing the gene expression related to osteocytic osteolysis and osteoclastic bone resorption; in males, it increases the gene expression related to steroid and fatty acid metabolism, thus protecting bones from bone resorption.^255^

The interaction between lipid metabolism and the cytokine signaling pathway is crucial for maintaining the balance of bone tissue cells such as osteoblasts, osteoclasts, and osteocytes. Under normal circumstances, the synergistic effect of the two ensures the dynamic balance of bone formation and bone resorption, maintaining bone homeostasis. If this interaction is imbalanced, for example, abnormal lipid metabolism leads to dysregulation of cytokine secretion, or abnormal cytokine signaling pathways affect lipid metabolism, it may disrupt the balance among bone cells, leading to problems such as bone mass loss or abnormal bone remodeling.

### The impact of lipid-mediated epigenetic regulation on bone homeostasis

#### Histone acetylation affected by lipid metabolism and osteoimmunological regulation

Acetyl-CoA produced by fatty acid β-oxidation is an important substrate for histone acetylation. When lipid metabolism is disordered (such as a high-fat diet), changes in the level of acetyl-CoA can affect the activity of histone acetyltransferases (such as p300) or deacetylases (such as SIRT1), thus participating in the regulation of macrophages and further influencing the regulation of bone homeostasis. As an NAD⁺-dependent deacetylase, SIRT1 is susceptible to suppression under high-fat-diet conditions. High-fat-diet-induced depletion of cellular NAD⁺ dampens SIRT1 activity, elevates histone H3K9 acetylation, and alleviates SIRT1-mediated repression of NF-κB. This cascade consequently upregulates the transcription of pro-inflammatory genes (e.g., TNF-α, IL-6) and drives macrophage polarization toward the pro-inflammatory M1 phenotype.^256,257^ Conversely, IL-4 activates SIRT3, which can deacetylate and activate GLUD1, promoting the decomposition of glutamine to generate αKG. αKG promotes the expression of M2-related genes by demethylating H3K27, ultimately driving the M2 polarization of macrophages.^258^ In contrast, ω-3 PUFAs such as EPA and DHA activate the AMPK/SIRT1 pathway, promote SIRT1-dependent deacetylation of the NF-κB p65 subunit, and suppress inflammatory cytokine expression in macrophages.^259^ In parallel, DHA activates PPARγ-mediated ERK/AKT signaling and promotes an M2-like microglial phenotype with increased IL-10 expression.^260^ IL-10 can inhibit the proliferation of osteoclast precursor cells, while TGF-β can promote the differentiation of osteoblasts. Compared with UFAs, the metabolism of SCFAs-related substances also plays an important role in the functional regulation of macrophages. Low-concentration butyric acid can increase the acetylation of H3K27 in the H19 promoter region by inhibiting HDAC2, promote the expression of H19 and the secretion of MMP15, and induce the polarization of M2 macrophages.^261^ Sodium butyrate (NaBu) can significantly inhibit the inflammatory response. By inhibiting HDACs, it enhances the acetylation level of p65 and reduces the expression and secretion of pro-inflammatory cytokines, thus inducing cells to exhibit an M2-like wound-healing phenotype. At the same time, it induces apoptosis of pro-inflammatory macrophages.^124^ In addition, the drug berberine can reduce the expression level of p300, weaken the binding of p300 to p65 Lys310, thereby inhibiting the acetylation level of p65 Lys310, resulting in a decrease in the translocation of p65 from the cytoplasm to the nucleus, further reducing the DNA-binding activity of NF-κB, and thus inhibiting the transcription of inflammatory genes^262^ (Table 2).

**Table 2:** The impact of lipid-mediated epigenetic regulation on bone homeostasis

Epigenetic-related substances	Lipid molecules	Epigenetic modification regulation Regulation of lipid metabolism	Effects on macrophages	Types of epigenetic modifications	Ref.
SIRT1	—	Lipid content ↑, SIRT1 activity ↓, histone H3K9 acetylation level ↑	NF-κB ↑, Pro-inflammatory gene expression ↑, Macrophages polarize towards the pro-inflammatory M1 type	Histone acetylation	^256,257^
	ω-3 PUFAs	Activate AMPK/SIRT1 and promote SIRT1-dependent deacetylation of NF-κB p65	Suppress LPS-induced inflammatory cytokine expression in macrophages	Histone deacetylation	^259^
HDACs	SCFAs	Inhibit HDAC2, H3K27 acetylation in the H19 promoter region ↑	Macrophages polarize towards the anti-inflammatory M2 type	Histone deacetylation	^261^
	NaBu	Inhibit HDACs, The acetylation level of p65 ↑	Expression and secretion of pro-inflammatory cytokines ↓, Suppress the inflammatory response, Macrophages polarize towards the anti-inflammatory M2 type	Histone acetylation	^124^
—	Berberine	The expression level of p300 ↓, Acetylation level of Lys310 at p65 ↓	Translocation of p65 from the cytoplasm to the nucleus ↓, Inhibit NF-κB, Suppress the transcription of inflammatory genes	Histone acetylation	^262^
αKG	—	H3K27 demethylation	Expression of M2-related genes ↑, Macrophages polarize towards the anti-inflammatory M2 type	Demethylation	^258^
METTL3	—	METTL3 catalyzes the m⁶A methylation modification of PTX3 mRNA ↑	PI3K/AKT and JAK/STAT6 signaling ↓, M2 macrophage polarization ↓	Methylation	^265^
DNMT1	—	CD36 ↓ ; ABCA1 and SOAT1 ↑ ;cholesterol accumulation ↓	Macrophage motility ↑; wound healing ↑	DNA methylation	^266^
HIF-2α	—	Regulate H3K27me3 at the Cpt1a promoter and suppress CPT1A-dependent fatty acid oxidation	NLRP3 inflammasome activation ↓; inflammatory response ↓	Histone methylation	^267^
Histone Methyltransferase (SETDB1)	—	Histone H3K9 methylation ↑, PPARγ activity ↓	M2 macrophage polarization ↓	Methylation	^268^
DNA Methyltransferase (DNMT)	5Aza	DNMT activity ↓, p65 phosphorylation ↑, IL-6 expression ↑, Combined with ABCA9, cholesterol efflux ↓, Cholesterol accumulation in macrophages	Macrophages polarize towards the pro-inflammatory M1 type	Methylation	^269^
—	ZOL	The m⁶A methylation level of NFATc1 (a key transcription factor for osteoclast differentiation) in osteoclasts ↑	Osteoclast differentiation ↓	Methylation	^270^
Lactic Acid	—	The lactylation level of PKM2 ↑, PKM2 activity ↑	M2 macrophage polarization ↑	Lactylation	^271^
MPC1	—	MPC1 knockout, K673 lactylation of fatty acid synthase ↓, Lipid accumulation ↓	Inflammatory level ↓, M2 macrophage polarization ↑	Lactylation	^272^
—	—	Lactylation of methyl-CpG binding protein 2 (MeCP2) at K271 ↑	M2 macrophage polarization ↑	Lactylation	^273^
—	—	STAT3 Palmitoylation ↓, Expression of c-Fos/NFATc1 ↓	Osteoclast differentiation ↓	Palmitoylation	^275^
Depalmitoylase	Phenylpyruvic Acid	Combined with PPT1, the activity of depalmitoylating enzyme ↓, Palmitoylation of NLRP3 protein ↑	Activation of NLRP3 inflammasome ↑, Release of inflammatory factors ↑	Palmitoylation	^276^

“—” indicates that no specific substance or lipid molecule is mentioned in the corresponding epigenetic regulation category

#### Lipid metabolism-mediated methylation modification and regulation of bone-related cell fate

Histone methylation or DNA methylation can be affected by lipid metabolism. Mevalonate, an intermediate in cholesterol synthesis, can affect the localization and function of epigenetic regulatory proteins (such as histone methyltransferases) by regulating isoprenylation modification.^263,264^ Research has found that METTL3 can inhibit the polarization of M2 macrophages by catalyzing the m⁶A methylation modification of PTX3 mRNA and suppressing the PI3K/AKT and JAK/STAT6 signaling pathways.^265^ DNMT1-dependent DNA methylation links lipid handling to macrophage motility: myeloid Dnmt1 deficiency reduces cholesterol accumulation, downregulates CD36, upregulates ABCA1 and SOAT1, and improves macrophage migration and wound healing. Separately, macrophage HIF-2α regulates H3K27me3 at the Cpt1a promoter and suppresses CPT1A-dependent FAO, thereby limiting NLRP3 inflammasome activation.^266,267^ In the non-canonical Wnt signaling pathway, the histone methyltransferase SETDB1 is activated. SETDB1 inhibits the trans-activation of PPARγ through histone H3K9 methylation of target genes.^268^ Meanwhile, the study also found that 5-aza-2’-deoxycytidine (5Aza), as an inhibitor of DNA methyltransferase (DNMT), can promote the phosphorylation of p65 and the expression of IL-6, bind to ABCA9, inhibit its cholesterol efflux function, lead to the accumulation of cholesterol in macrophages, and further activate M1-like macrophages.^269^ In addition, in terms of pharmacology, for bisphosphonates (such as zoledronic acid, ZOL), first-line drugs for osteoporosis treatment, the m⁶A methylation level of NFATc1 (a key transcription factor for osteoclast differentiation) in osteoclasts is significantly increased, inhibiting osteoclast differentiation and its function^270^ (Table 2).

#### Other epigenetic modification methods: the roles of lactylation and palmitoylation in osteoclast regulation

In addition to acetylation and methylation, there are also various epigenetic modification methods in cells, such as lactylation and palmitoylation. Besides inhibiting the Warburg effect by activating PKM2 and promoting the transition of pro-inflammatory macrophages to a repair phenotype, lactate can also regulate cellular life activities by increasing the lactylation of multiple proteins. For example, pyruvate kinase M2 (PKM2) is an important molecular determinant for the metabolic adaptation of pro-inflammatory macrophages. As a substrate for lactylation, lactate can increase the lactylation level of PKM2, inhibit the conversion of its tetramer to dimer, enhance pyruvate kinase activity, and regulate the transition of the inflammatory phenotype of macrophages.^271^ In addition, in high-fat diet-fed mice, MPC1 knockout can affect the lactylation process of multiple proteins by regulating the lactate level in hepatocytes, especially the inhibitory effect of lactylation at the K673 site of fatty acid synthase. This mediates the downregulation of lipid accumulation, changes in macrophage polarization, and a reduction in the inflammatory level.^272^ Meanwhile, clinical research also found that lactylation of methyl-CpG binding protein 2 (MeCP2) at K271 can be observed in plaque macrophages. This modification promotes the polarization of pro-repair M2 macrophages, reduces the plaque area, shrinks the necrotic core, decreases plaque lipid deposition, and increases collagen content.^273^ In addition, lactate can enter macrophages through monocarboxylate transporters (MCTs), rely on p300/CBP to catalyze the lactylation of HMGB1, and be released through exosomes, thus disrupting the endothelial barrier.^274^ Compared with lactylation, palmitoylation is mainly involved in signal transduction pathways and the regulation of inflammasomes. During the process of receptor activator of RANKL-induced osteoclastogenesis, disrupting STAT3 palmitoylation inhibits RANKL-induced c-Fos/NFATc1 expression, thereby suppressing osteoclastogenesis.^275^ For the inflammasome, after phenylpyruvic acid is taken up by macrophages, it binds to PPT1, inhibiting the activity of depalmitoylase, thus enhancing the palmitoylation modification of NLRP3 protein, promoting the activation of the NLRP3 inflammasome, and the release of inflammatory factors such as interleukin (IL)-1β^276^ (Table 2).

## Therapeutic insights and application prospects

### Therapeutic approaches modulating macrophage lipid metabolism in bone remodeling

**Fig. 7:**
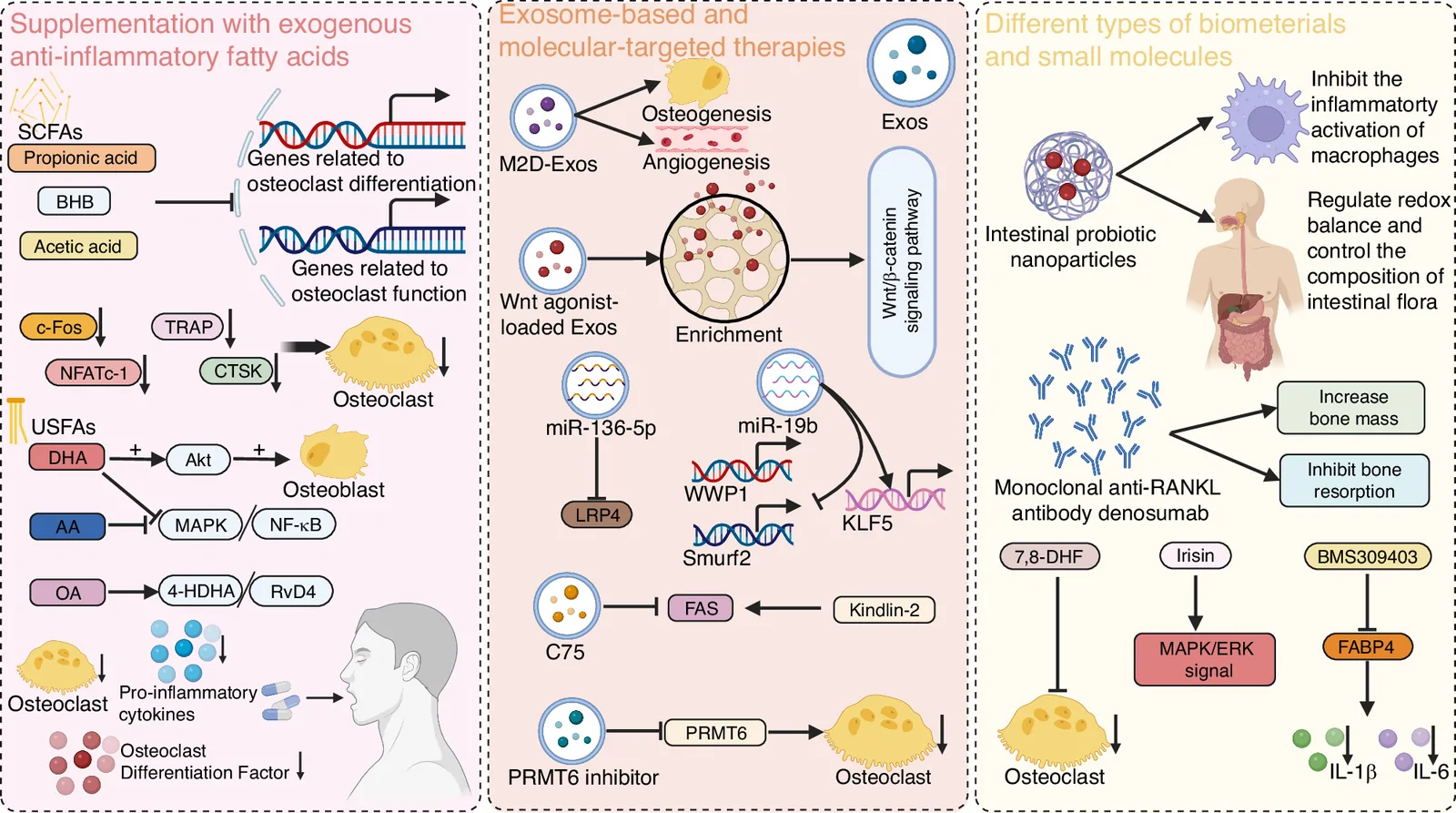
Therapeutic strategies related to bone metabolism. Supplementing exogenous anti-inflammatory fatty acids: short-chain fatty acids inhibit osteoclast differentiation/function-related genes; unsaturated fatty acids promote osteogenesis through Akt, and reduce pro-inflammatory cytokines and osteoclast differentiation factors. Exosome therapy: M2D-Exos promote osteogenesis and angiogenesis; some exosomes regulate genes through the Wnt/β-catenin pathway or via miRNAs and inhibitors (such as PRMT6 inhibitors, C75, etc.), thereby inhibiting osteoclasts. Biomaterials and drugs: intestinal probiotic nanoparticles inhibit the inflammatory activation of macrophages and regulate intestinal flora; denosumab increases bone mass and inhibits bone resorption

The impact of macrophage lipid metabolism on fractures mainly depends on the differentiation of osteoclasts and osteoblasts. During the differentiation process, some lipid molecules can inhibit the differentiation of osteoclasts or promote the formation of osteoblasts, thus achieving the effect of promoting fracture healing (Fig. 7).

#### Anti-inflammatory fatty acids and ketone bodies

Among various lipid substances, SCFAs have been repeatedly proven to be able to participate in the differentiation process of osteoclasts, achieving the effect of promoting fracture healing. Among them, the ketone body β-hydroxybutyric acid (BHB) inhibits the inflammasome independently of the Gpr109a receptor and HDAC inhibition. In a dose-dependent manner, it inhibits the expression of osteoclast differentiation-related genes (TRAF6, NFATc-1, c-Fos) and function-related genes (CTSK, MMP9, TRAP) by inhibiting HDAC, and reduces the formation of F-actin rings.^277^ Meanwhile, the study found that consuming water or food containing BHB precursor 1,3-butanediol can increase the circulating BHB level, reduce bone loss in the mouse osteolysis model, and decrease the number of osteoclasts.^278^ In addition, SCFAs (mainly acetic acid and propionic acid) can inhibit the release of cytokines that promote osteoclastogenesis, thereby inhibiting osteoclast formation, reducing bone resorption, promoting bone formation, and bone metabolism balance.^279^

In addition, PUFAs corresponding to SCFAs also play an important regulatory role in the differentiation of osteoclasts. Unsaturated fats can inhibit the differentiation of osteoclasts. Therefore, in dietary adjustments or adjuvant drug therapies, the supplementation of PUFAs is also a way to accelerate fracture healing. Among them, ω-3 PUFA (such as DHA) can not only enhance the Akt signal by remodeling the plasma membrane lipidome to induce stable microdomains, thereby specifically promoting the osteogenic differentiation of MSCs and inhibiting adipogenic differentiation,^280^ but also reduce various lipid levels in the plasma. By reducing the activity of the insulin and inflammatory signaling pathways in HSCs, it decreases the expression of bone resorption markers, increases the content of DHA and EPA in the bone marrow, and improves the bone microenvironment. This improves the osteogenic differentiation ability of BMSCs, reduces adipogenic differentiation and senescent phenotypes, and inhibits the differentiation of OCs.^281^ Docosahexaenoic acid (DHA) and AA may inhibit the NF-κB and MAPK signaling pathways downstream of RANKL, reduce the expression of pro-inflammatory cytokines (such as IL-6, TNF-α) and osteoclast differentiation factors (such as DC-STAMP), and significantly inhibit osteoclastogenesis. In addition, the metabolite of DHA, Resolvin D1, can directly inhibit osteoclast fusion, while the metabolite of AA, lipoxin A4, has anti-inflammatory and anti-bone resorption effects. Dietary supplementation with DHA or AA may inhibit excessive osteoclast activation.^282^ In addition, monounsaturated fatty acids (MUFAs) like oleic acid (OA) and palmitic acid (PA) can affect the differentiation of osteoclasts by regulating the signaling pathway through RvD4 and 4-HDHA. Among them, PA enhances the inflammatory response stimulated by lipopolysaccharide (LPS) and promotes the differentiation of osteoclast precursors, while OA can inhibit the occurrence of this reaction.^283^

#### Exosome-based delivery of lipid-metabolism modulators

Exosomes (Exos), as small extracellular vesicles, are important paracrine carriers in non-cell therapy.^284^ Exosomes can carry numerous cellular secretions as well as various inhibitors or activators. For example, exosomes derived from M2 macrophages (M2D-Exos) enhance the proliferation and migration abilities of human bone marrow mesenchymal stem cells (HMSCs) and human umbilical vein endothelial cells, and promote osteogenesis and angiogenesis both in vivo and in vitro.^285^ Wnt agonist-loaded Exos can accumulate in bones after intravenous injection.^286^ Exosome-delivered Wnt agonists can activate the Wnt/β-catenin pathway and participate in regulating lipid metabolism in macrophages and the differentiation of osteoblasts and osteoclasts.

Exosomal miRNAs affecting lipid metabolism and osteogenesis can target specific cells and act on genes in an aggregated manner to regulate gene expression. For example, miR-19b in BMSCs inhibits the expression of WWP1 or Smurf2 and increases the expression of KLF5 through the Wnt/β-catenin signaling pathway, thus promoting fracture healing.^287^ MicroRNA-136-5p inhibits the expression of low-density lipoprotein receptor-related protein 4 (LRP4), activates the Wnt/β-catenin signaling pathway, and accelerates the process of fracture healing.^288^ In addition to microRNAs, PRMT6 is a key metabolic checkpoint. During osteoclast differentiation, it inhibits FAO and promotes HIF-1 signaling-dependent glycolysis by regulating the asymmetric dimethylation of H3R2 at the promoters of key genes, achieving metabolic conversion and supporting osteoclast differentiation and activation. Inhibiting PRMT6 can reduce osteoclastogenesis and bone loss, making it a promising target for anti-osteoporosis treatment.^289^ Kindlin-2 in adipocytes can stabilize FAS, activate the PPARγ/FABP4 pathway, inhibit insulin secretion, and thus suppress bone formation. Inhibiting Kindlin-2 can degrade FAS, reduce FABP4, release insulin, and promote osteogenesis.^290^ Accordingly, in terms of drug use, FAS inhibitors (such as C75) or Kindlin-2 targeted RNA editing (AAV vector) can be used as new treatments for osteoporosis. However, C75 has obvious side effects, and further research is needed.

#### Biomaterials and nanoparticle systems enhancing SCFA production

In the human body, intestinal absorption is the main source of SCFAs. Besides additional supplementation of SCFAs, increasing the concentration of SCFAs can also promote fracture healing. Recently, it has been newly discovered that extracellular vesicles (mEVs) and nanotherapies can participate in the regulation. For example, recent research has found that milk-derived extracellular vesicles (mEVs) can reshape the gut microbiota,^291^ increase the concentration of SCFAs, and reduce the level of cytokines that promote osteoclastogenesis. In terms of bone formation, mEVs can increase indicators such as bone volume and bone density of the distal femur, reduce trabecular bone spacing, improve the situation of adipocytes in the bone marrow, inhibit osteoclast differentiation both in vivo and in vitro, alleviate the symptoms of osteoporosis, and are expected to become a food supplement for the prevention and treatment of osteoporosis.^292^ In addition, the research found that many intestinal probiotic nanoparticles, such as shellac resin, can achieve the slow release of probiotic butyric acid in the colorectal region for polyvinyl butyral nanoparticles, effectively inhibit the inflammatory activation of macrophages, regulate the redox balance, and regulate the composition of the gut microbiota.^211^

#### Small-molecule, antibody and gene regulatory drugs

In the field of small-molecule therapy, in addition to specific administration methods, there are numerous targeted drugs that can be directly injected or used. Research has found that a monoclonal anti-RANKL antibody, denosumab, can inhibit bone resorption, increase bone mass, and reduce the risk of fractures. However, discontinuation of denosumab can trigger a widespread wave of rebound bone resorption through the RANKL/OPG pathway.^293^ ELOVL6 in macrophages can also affect lipid absorption or efflux,^294^ inhibiting ELOVL6 can reduce the enhancement of ABCA1-mediated lipid efflux through the S1P/PPARγ pathway, decrease the expression of inflammatory factors, present a reparative phenotype, and promote fracture healing. PPARα also participates in lipid-sensitive control of macrophage phenotype. In LPS-stimulated macrophages, CYP2J2-derived epoxyeicosatrienoic acids suppress NF-κB signaling and M1 polarization through PPARα/γ- and HO-1-dependent mechanisms.^295^ For example, butyric acid can inhibit NF-κB-mediated M1-like polarization by activating PPARα.^296^ These findings support an anti-inflammatory role for the butyrate–PPARα axis in colonic macrophages, although direct evidence that this mechanism promotes fracture healing remains to be established. Targeting enzymes related to lipid metabolism can also participate in regulating the polarization of macrophages and contribute to the treatment of bone diseases. In addition to antibody-based drugs, some gene regulators can also be directly injected or taken orally. For example, the FABP4 inhibitor BMS309403 can significantly reduce the expression of pro-inflammatory factors (such as IL-1β, IL-6, etc.) by inhibiting the expression of FABP4, and decrease the number of apoptotic cells.^297^ In addition, other small molecules also play important roles. For example, Irisin may promote the expression of genes related to osteogenesis and angiogenesis by activating the MAPK/ERK signaling pathway, enhance osteogenic differentiation, and promote angiogenesis, thus accelerating fracture healing.^298^ In addition, Irisin can also activate the AMPK pathway by binding to Caveolin-1, promote osteoblast proliferation, and inhibit ferroptosis through the Nrf2/HMOX1/Fpn axis.^299^ The plant-derived small molecule 7,8-dihydroxyflavone (7,8-DHF) also has a significant regulatory effect on bone metabolism. It has the ability to dual-regulate the osteogenic and osteoclastic processes and improve bone loss. 7,8-DHF can activate the Wnt/β-catenin pathway, upregulate Runx2/Osterix, and promote osteoblast function dependent on the TrkB receptor. At the same time, 7,8-DHF can upregulate OPG mRNA, reduce RANKL expression, increase the OPG/RANKL ratio, and inhibit the activation of the NF-κB pathway, thereby inhibiting osteoclast differentiation. Thus, it achieves a dynamic balance of bone metabolism through dual regulation.^300^

### The application potential of lipid therapy in inflammatory bone diseases

Inhibiting cholesterol synthesis, removing extracellular lipids, and promoting cholesterol efflux can all inhibit LPS-induced pro-inflammatory gene expression, which has become an important direction for the treatment of inflammatory bone diseases. Desmosterol is an important LXR activator and a negative regulator of inflammasome activation in macrophages. When desmosterol in macrophages decreases, LXR/RXR is inhibited, and the expression of ABCA1 and ABCG1 is reduced, leading to the accumulation of neutral lipids such as cholesterol and the exacerbation of inflammatory responses.^301^ Therefore, the targeted regulation of desmosterol is expected to become a therapeutic strategy for inflammatory bone diseases. However, research on desmosterol is still incomplete. Meanwhile, studies have found that polyrotaxane (PRX) can activate LXR, promote the expression of ABCA1 and cholesterol efflux, thereby inhibiting the inflammatory activation of macrophages.^302^ In addition, it was found that rice bran active peptide (RBAP) can increase the levels of factors related to reverse cholesterol transport (RCT) in macrophages through a TLR4-dependent manner, promote the transfer of cholesterol from macrophages to HDL or apoA1, and regulate the TLR4/MyD88/NF-κB signaling pathway to inhibit inflammatory responses.^303^ Accordingly, targeting cholesterol metabolism is an effective strategy for inflammatory bone diseases.

Scholars have conducted a large amount of research and proposed many potential treatment directions regarding the anti-inflammatory effects of UFAs and the pro-inflammatory effects of SFAs. Long-chain SFAs can dose-dependently upregulate the expression of Egr2 in bone marrow-derived macrophages, thereby increasing intracellular neutral lipids and promoting the pro-inflammatory differentiation of macrophages. In contrast, UFAs can inhibit the SFA-induced expression of Egr2.^304^ Accordingly, reducing the intake of SFAs in daily life and reasonably combining UFAs can play a certain role in alleviating the occurrence of inflammation. Fatty acid-binding protein 5 (FABP5) can mediate the cellular uptake of UFAs, but in cancer patients, the expression of FABP5 can cause immunosuppression.^305^ In NAFLD models, macrophage-derived itaconate acts on hepatocytes to reduce lipid accumulation and enhance FAO-dependent oxidative phosphorylation, while 4-OI reverses high-fat-diet-associated dyslipidemia. In parallel, activation of the macrophage Irg1/itaconate axis enhances phagocytosis and improves outcomes in models of intracerebral hemorrhage and peritonitis.^306,307^ These findings highlight the context-dependent therapeutic potential of the itaconate pathway.

In addition to long-chain fatty acids, SCFAs also play an important role in the metabolic regulation of macrophages. Propionic acid (C3) and butyric acid (C4) can inhibit pyroptosis after the activation of the NLRP3 inflammasome and osteoclast formation stimulated by IL-1β, reducing bone erosion pits.^308^ SerBut, a drug synthesized by conjugating butyrate with L-serine (L-Ser), can significantly improve the oral bioavailability of butyrate, increase the M2/M1 macrophage ratio, and serve as a potential new therapeutic agent. However, further preclinical and clinical studies are still needed to clarify its long-term safety and efficacy.^309^

Palmitic acid is an important component of cell energy and is involved in various metabolic pathways. Targeting the synthesis and metabolism of palmitic acid can regulate the polarization of macrophages, providing a therapeutic strategy for inflammatory bone diseases. FASN catalyzes the production of palmitic acid, which palmitoylates Cys898 of NLRP3, thereby activating the NLRP3 inflammasome.^310^ In addition, elevated serum palmitic acid levels can activate the macrophage TLR4/ERK/FOXC2 pathway, upregulate Dll4 expression, activate the Notch signaling pathway in VSMCs, and induce M1 polarization of macrophages.^311^ Accordingly, inhibiting FASN or inhibiting the synthesis of palmitic acid provides a solution for suppressing inflammatory diseases, but the specific mechanism of action in macrophages and the corresponding enzymes are still not very clear. However, different from the above two types of palmitic acid metabolism, palmitic acid-modified bovine serum albumin (PAB) nanoparticles can target and activate scavenger receptor-A (SR-A) on macrophages, effectively treating rheumatoid arthritis (RA).^312^ Therefore, palmitoylation of different substances can produce different effects.

In addition to the above treatments for the impact on fatty acids in the package, gene therapy and exogenous substance supply to regulate the lipid metabolism process of macrophages are also important treatment options.

Research has found that fat-1 gene therapy can reduce the level of PGE2, increase the level of PGE3, lower the serum ω-6:ω-3 FA ratio, and at the same time increase the content of anti-inflammatory macrophages, thereby significantly reducing the severity of OA and improving bone microstructure.^313^ In terms of lipid uptake, some studies have found that Atf3 activates the AMP-activated protein kinase α (Ampkα) signaling pathway to promote FAO.^314^ Therefore, targeted treatment of Atf3 is expected to be applied to bone diseases. In terms of exogenous substance supply, research has found that in the microenvironment of patients with obesity-related osteoarthritis (OA), the blood lipid level increases, leading to an increase in M1 macrophage infiltration, a downregulation of the secretion of growth arrest-specific protein (GAS6), and an impairment of the phagocytic ability of macrophages towards apoptotic cells. This causes hyperplasia of the synovial tissue and ultimately leads to the inflammatory progression of osteoarthritis. However, intra-articular injection of GAS6 can improve the symptoms^315^ (Fig. 7).

### Challenges and opportunities from basic research to clinical application

Lipidomics—particularly redox lipidomics/phospholipidomics—can provide mechanistic insight into lipid oxidation in the marrow space by directly detecting and quantifying oxidized lipid products, including oxidized phospholipids (OxPLs) and oxidatively truncated derivatives. These species constitute molecular “fingerprints” of lipid peroxidation and offer pathway-relevant information beyond bulk ROS measurements. In ferroptosis biology, LC–MS-based redox lipidomics has resolved defined oxygenated phosphatidylethanolamine (oxPE) signatures, including lipoxygenase-associated hydroperoxy-PE intermediates, supporting the concept that product-level structural information can help distinguish regulated (enzyme-associated, regioselective) peroxidation from broader free-radical chain oxidation patterns.^316–318^

Within the bone marrow niche, marrow adipocytes/BMAT can enrich local PUFA-containing lipid pools and thereby increase susceptibility to phospholipid peroxidation; for example, marrow adipocyte–derived extracellular vesicles can transfer lipid droplets to osteoblasts, increasing intracellular PUFA availability and promoting ferroptosis-linked injury.^319^ Mechanistically, lipidomic readouts integrated with genetic or pharmacologic perturbation can link oxidation to key control nodes such as GPX4,^320^ and spatial lipidomics (e.g., MALDI–MS imaging) provides a route to localize lipid remodeling across bone-marrow compartments.^321,322^ Given the chemical lability of oxidized lipids, rigorous sample handling and standardized protocols remain critical to minimize ex vivo artifacts when interpreting marrow oxidized-lipid profiles.^323^

In the clinical translation of research findings on lipid metabolism, some studies demonstrate promising application prospects for anti-inflammation and anti-bone loss. However, it has a certain tumor-promoting effect on cancer patients, and the risk to healthy people remains unknown. The assessment of risks and the clinical improvement of cancer patients still need to be enhanced. Context-dependent effects must be considered when translating macrophage lipid-targeted therapies. In tumor-associated macrophages, lactate/acidic pH and IL-4/IL-13 can induce CH25H through MCT1- and STAT6-dependent mechanisms. CH25H-derived 25HC accumulates in lysosomes, engages the GPR155–mTORC1–AMPKα axis, and reinforces immunosuppressive macrophage programs. Thus, inhibiting MCT1, STAT6, or CH25H may attenuate CH25H induction in tumors; however, these observations cannot be directly extrapolated to bone formation, and skeletal and oncologic effects require context-specific evaluation.^324^ However, recent research has identified the FABP4⁺C1q⁺ macrophage subset, which highly expresses FABP4 and C1q. This subset promotes the expression of pro-inflammatory cytokines through the AMPK/JAK/STAT signaling axis. Notably, FABP4⁺C1q⁺ macrophages are positively correlated with CD8⁺ T cell infiltration and can exert an anti-tumor effect by enhancing the anti-tumor immune microenvironment.^325^ The above reflects the relationship between the pro-inflammatory/anti-inflammatory effects and tumors. The anti-inflammatory effect, accompanied by reduced fatty acid metabolism, increases the risk of osteogenesis in tumor patients. However, the key regulatory factor AMPK has great research value. Its different effects lead to completely different results, and further research on it has the value of balancing the relationship.

In the dietary composition of people, SCFAs play an important regulatory role in the body’s immunity and also have an important regulatory effect on the polarization of macrophages.

However, deeper mechanistic investigations remain limited. SCFAs regulate the number and function of colonic regulatory T cells (Tregs) by activating G-protein-coupled receptor (GPCR) signaling (e.g., Ffar2) and inhibiting HDACs.^326^ SCFAs inhibit the inflammatory signaling pathway by activating GPCRs, reducing the production of pro-inflammatory cytokines and increasing the release of anti-inflammatory cytokines. At the same time, SCFAs can promote the fusion of phagosomes with lysosomes and enhance their phagocytic function, which helps to eliminate pathogens. In terms of macrophage polarization, SCFAs promote the polarization of macrophages from the pro-inflammatory M1 type to the anti-inflammatory M2 type, participating in the regulation of the balance between inflammation and tissue repair.^327^ Therefore, SCFAs have the potential to regulate macrophage function and enhance the immune response, and can be used as adjuvant therapeutic drugs for inflammatory diseases. However, further research is needed to clarify their mechanism of action and balance the therapeutic effect and risks. For example, among SCFAs, butyric acid can upregulate the expression of FPN in macrophages, promote iron export, and thus inhibit the production of pro-inflammatory cytokines in macrophages. However, the exploration of its mechanism of action in other aspects is relatively scarce. Moreover, the research on the corresponding fatty acids in a large number of SCFAs still has limitations.^328^

In addition to SCFAs, the most common focus in daily life still lies in SFAs and UFAs. For example, α-linolenic acid (ALA) can prevent inflammatory bone loss by inhibiting the NF-κB-iNOS-COX-2 signaling pathway, reducing osteoclastogenesis and the release of inflammatory factors. Therefore, ALA can be a potential drug for preventing inflammatory bone diseases such as osteoporosis and rheumatoid arthritis.^329^ However, the direct effect of ALA on osteoblasts has not been explored, and its long-term toxicity needs further verification.

In addition to the aspects of diet and tumor impact, most of the regulatory sites and small molecule signals in the regulatory mechanism of fatty acids still remain to be studied. For example, Janus kinase 2 (JAK2) deficiency can lead to impaired cholesterol efflux, exacerbating inflammatory responses. The LXR agonist T0901317, on the other hand, can increase cholesterol efflux in M-Jak2 KO macrophages.^330^ Therefore, JAK2 can serve as a potential therapeutic target for inflammatory diseases, but the mechanism of action of JAK2 in inflammatory bone diseases remains unclear. Furthermore, studies have also found that BHLHE40/41 may regulate LXR:RXR and MiT/TFE family transcription factors through negative feedback, affecting pathways related to cholesterol clearance and lysosomal processing. Knockout or knockdown of BHLHE40/41 can enhance the activity of these pathways and promote cholesterol efflux and storage. However, the specific mechanism remains unclear^331^ (Fig. 7).

## Conclusions and future prospects

In summary, lipid metabolism plays a central regulatory role in the dynamic balance between bone resorption and formation by precisely controlling macrophage polarization. Specifically, long-chain fatty acids and cholesterol accumulation tend to drive pro-inflammatory M1 polarization, exacerbating bone destruction. Conversely, SCFAs, PUFAs, and cholesterol efflux promote anti-inflammatory M2 polarization, thereby promoting the repair of inflammatory bone damage and bone formation after fractures. This understanding heralds a new era in treating bone diseases. Future research should focus on two primary frontiers. First, developing precision therapeutics that target macrophage lipid metabolism, such as probiotic-based strategies that modulate the “gut-bone axis” to indirectly reshape the bone immune microenvironment. Second, translating these fundamental insights into clinical practice through a multi-modal approach that integrates metabolic drugs, advanced biomaterials, and dietary interventions. This strategy promises comprehensive solutions for clinical challenges like fracture healing, osteoporosis, and inflammatory bone diseases, overcoming the limitations of current therapies and holding immense potential for revolutionizing bone health management.
